# Transcriptional Networks Controlling the Cell Cycle

**DOI:** 10.1534/g3.112.004283

**Published:** 2013-01-01

**Authors:** Martin Bonke, Mikko Turunen, Maria Sokolova, Anna Vähärautio, Teemu Kivioja, Minna Taipale, Mikael Björklund, Jussi Taipale

**Affiliations:** * Genome-Scale Biology Research Program, Institute of Biomedicine, Biomedicum Helsinki, University of Helsinki, Helsinki, Finland; † Department of Biosciences and Nutrition, Karolinska Institutet, Stockhom, Sweden; ‡ Department of Computer Science, University of Helsinki, Helsinki, Finland; § Division of Cell and Developmental Biology, College of Life Sciences, University of Dundee, Dundee, Scotland, UK

## Abstract

In this work, we map the transcriptional targets of 107 previously identified *Drosophila* genes whose loss caused the strongest cell-cycle phenotypes in a genome-wide RNA interference screen and mine the resulting data computationally. Besides confirming existing knowledge, the analysis revealed several regulatory systems, among which were two highly-specific and interconnected feedback circuits, one between the ribosome and the proteasome that controls overall protein homeostasis, and the other between the ribosome and Myc/Max that regulates the protein synthesis capacity of cells. We also identified a set of genes that alter the timing of mitosis without affecting gene expression, indicating that the cyclic transcriptional program that produces the components required for cell division can be partially uncoupled from the cell division process itself. These genes all have a function in a pathway that regulates the phosphorylation state of Cdk1. We provide evidence showing that this pathway is involved in regulation of cell size, indicating that a Cdk1-regulated cell size checkpoint exists in metazoans.

In organisms ranging from yeast to humans, differences in transcription factor activity during the cell cycle cause a significant fraction of all genes to be regulated periodically (Whitfield *et al.* 2002; Rustici *et al.* 2004). This cyclic transcription ensures that proteins required for different cell-cycle phases are produced at the appropriate time (Jensen *et al.* 2006). Proteins that regulated the previous phase are, in turn, often selectively targeted for degradation by the proteasome. The genes that are cyclically expressed encode proteins directly involved in DNA replication and cell division, and master regulators of the cell division process, such as the cyclins. The cyclins bind to and regulate the activity of cyclin-dependent kinases (Cdks) that control cell-cycle processes and activate transcription factors, which subsequently modulate expression of genes needed for the next phase of the cell cycle. In animal cells, this process is at least in part driven by the transcription factors E2f and Myb, whose activities are in turn regulated by Cdks (reviewed in Koepp *et al.* 1999; Kastan and Bartek 2004; Murray 2004).

The progression through the cell cycle can be halted at several checkpoints where completion of cell-cycle processes is monitored. The DNA damage checkpoint (Kastan and Bartek 2004) arrests the cell cycle in cells that have incurred DNA damage or have failed to replicate DNA completely. The spindle assembly checkpoint in turn prevents premature separation of sister chromatids in mitosis (Musacchio and Salmon 2007; Pesin and Orr-Weaver 2008). In addition, it has been suggested that the correct segregation of subcellular organelles, such as the Golgi apparatus, is also monitored by checkpoints (Colanzi and Corda 2007). Triggering of checkpoints results in the activation of repair and/or apoptotic processes and associated transcriptional responses that are not part of the normal cell cycle.

In somatic cells, progression through the cell cycle also requires cell growth. At the transcriptional level, cell growth is controlled by the transcription factor Myc (dm), which heterodimerizes with Max and regulates a large number of genes, including genes involved in ribosome biogenesis (Greasley *et al.* 2000; van Riggelen *et al.* 2010). Mutations in *Myc* or ribosomal genes lead to small body size in *Drosophila* (Kongsuwan *et al.* 1985; Marygold *et al.* 2007), indicating that cellular ribosome levels are rate limiting for growth.

Although cell division requires growth, the inverse is not true as cell growth does not require cell division. Classical analysis of cell division cycle yeast mutants revealed that cells prevented to undergo cell division due to loss of cyclin-dependent kinase activity continue to synthesize proteins and grow to a larger size (Hartwell *et al.* 1973; Nurse *et al.* 1976; Reed 1980; Dickinson 1981; Goranov *et al.* 2009). Similarly, arrested *Drosophila* imaginal disc cells grow to a very large size, but pattern relatively normally (Weigmann *et al.* 1997), which indicates that cell size is controlled independently of tissue size. The mechanisms that control cell size in the unicellular organism *S. pombe* are relatively well understood (Martin and Berthelot-Grosjean 2009; Moseley *et al.* 2009; Hachet *et al.* 2011). However, the mechanisms that regulate metazoan cell size under physiological conditions remain unknown. Some experiments in mammalian cells support a model in which cell division is controlled independently of cell size, and that cell size is maintained by a passive mechanism (Conlon and Raff 2003). In this model, the rate of cell growth is independent of cell size, and the linear increase in cell size is counteracted by exponential decrease of size caused by division. Other experiments, however, indicate that large cells grow faster, suggesting that cell size must be controlled actively (Dolznig *et al.* 2004; Tzur *et al.* 2009; Park *et al.* 2010). Furthermore, recent research shows a close link between cell size, cell growth, and the cell cycle in mammalian cells (Son *et al.* 2012), indicating that a cell-size control mechanism is indeed present. The mechanism by which such an active cell size control mechanism would operate is completely unknown. We believe our data have now uncovered at least part of this checkpoint.

We used *Drosophila* Schneider cell line 2 [S2; (Schneider 1972)] in our analysis because *Drosophila* is the simplest widely used model organism whose cell-cycle regulation is similar to that of vertebrates. Also, its genome is relatively small and lacks much of the redundancy observed in higher organisms. Although the cell-cycle regulation during early embryogenesis differs significantly between *Drosophila* and mammals, regulation of cell growth and the cell cycle of somatic cells is very similar [reviewed in (Koepp *et al.* 1999; Kastan and Bartek 2004; Murray 2004)]. The availability of genome-wide expression arrays together with the very high efficiency of RNA interference (RNAi) makes *Drosophila* a good model to map transcriptional regulatory interactions during the cell cycle.

We previously identified ∼80% of the genes required for normal cell-cycle progression in *Drosophila* (Bjorklund *et al.* 2006). In this work, we map the transcriptional targets of the genes whose loss induced the strongest effects by using RNAi followed by whole-genome expression profiling. By treating the resulting expression profiles as phenotypes (see Holstege *et al.* 1998; Hughes *et al.* 2000), we were able to identify genes that act upstream of or at the level of the direct transcriptional regulators, and genes that are coregulated across RNAi treatments. Our results reveal several regulatory circuits, including a feedback circuit that includes Myc/Max, the ribosome, and the proteasome. Furthermore, we find that the cell-division process and the cyclic transcriptional program, controlled by the cyclin-dependent kinases and by E2f and Myb, respectively, can be disconnected from each other by treatments that positively affect Cdk1 (*cdc2*) activity. All such treatments resulted in decrease in cell size, whereas negatively affecting Cdk1 activity by knocking down its phosphatase Cdc25 (*string*) has the opposite effect. We also find that cellular levels of unphosphorylated Cdk1 increase exponentially in relation to cell size, suggesting a role for this mechanism in the regulation of cell size.

## Materials and Methods

### Cell culture, double-stranded RNA (dsRNA) generation, and transfection

*Drosophila* S2 cells (Schneider 1972) were cultured at controlled temperature (+23.5°; Binder KB cooling incubator) in Invitrogen SFM medium supplemented with 10% fetal bovine serum, 10% IMS (insect medium supplement; Sigma-Aldrich), 2 mM glutamine, and antibiotics.

The templates for the dsRNAs were generated by polymerase chain reaction (PCR) from either specific *Drosophila* gene collection clones, or from full-length, sequence-verified cDNA templates derived from SD Schneider cell cDNA library (Berkeley *Drosophila* Genome Project) that were cloned into pMAGIC1 [(Li and Elledge 2005) see Supporting Information, Table S10 for primer sequences). Standard oligos containing the 5′ T7 RNA polymerase recognition sequence were designed for these vectors and used with the Megascript kit (Ambion) according to manufacturer’s instructions to generate the dsRNAs used for the transfections.

For transfections in 6-well plates, 10^6^ S2 cells were seeded per well in 2 mL of medium. The following day, cells were transfected with dsRNAs using Effectene (QIAGEN) according to the manufacturer’s instructions, except that per well, 1.1 µg of dsRNA, 8.8 µL of enhancer, and 5 µL of Effectene in EC buffer (125 µL of final volume) was used. Experiments were performed in quadruplicate (four biological replicates) for genes that are expected to have direct impact on transcription (the transcription factors), and in singlicate for genes whose effect on transcription is expected to be indirect via the TFs. To counter the batch effect, each set of transfections also contained four control transfections with dsRNA targeting the green fluorescent protein (GFP) gene. Human U2OS osteosarcoma cells were cultured in GlutaMax media (Gibco) supplemented with 10% fetal bovine serum and antibiotics.

### RNA-sequencing (RNA-seq)

RNA-seq libraries were prepared as previously described (Kivioja *et al.* 2011) with the following changes: 1.8 μg of each RNA sample was used for cDNA synthesis with a modified template switch oligo (5′-ACACTCTTTCCCTACACGACGCTCTTCCGATCT-(5 base barcode)-rGrGrG-3′; Eurofins MWG Operon) and libraries were amplified using Advantage 2 PCR system (Clonetech) with 16 cycles of a two-step program. Sequences were obtained with Illumina GA IIx and HiSeq sequencers and mapped to transcripts as described in Kivioja *et al.* (Kivioja *et al.* 2011). Samples containing more than one million reads were used in the clustering analyses. The original data are submitted to ArrayExpress and will be made available (E-MTAB-1364).

### Flow cytometry and RNA extraction

After transfection, the S2 cells were cultured for 4 d and subsequently collected by agitation and centrifugation. The culture media was removed, and the cells resuspended in 1 mL of ice-cold phosphate-buffered saline (PBS). A 25-μL sample was then taken and fixed overnight with 70% ethanol at −20°. The remainder of the cell suspension was centrifuged again, the PBS removed, and the cell pellet stored at −80° until RNA extraction was performed. After overnight fixation, the ethanol of the 25-μL aliquot was replaced with PBS containing 30 µg/mL propidium iodide (Molecular Probes P1304MP) and 30 µg/mL RNAse A (Sigma R5125). The cells were stained for 30 min at +37°, after which flow cytometry analysis was performed using a FACSArray flow cytometer (Beckton Dickinson). Flow cytometry graphs were then analyzed using FACSDiva software (Beckton Dickinson, FACSArray). RNA extraction was performed with QIAGEN RNeasy kits according to manufacturer’s instructions and included the optional DNase treatment.

For flow cytometric analysis of total Cdk1 and Y15-phospho-Cdk1 in U2OS cells, a mouse−rabbit and rabbit−mouse antibody pairs were used in such a way that fluorescence channels and secondary antibodies were reversed between the experiments to rule out artifactual signals. Cells were fixed with cold 70% ethanol and left at −20° overnight. The cells were then washed with 1% bovine serum albumin (BSA) in PBS and blocked in 1% BSA in PBS for 30 min. Cells were incubated with the phospho-specific Cdk1 antibody (CellSignaling #9111 rabbit polyclonal Ab and ECM Biosciences CM2311 mouse monoclonal in Exp#1 and Exp#2, respectively) overnight, washed three times, and then incubated with the total Cdk1 antibody (CellSignaling #9116 mouse monoclonal and Santa Cruz Biotechnology sc-53 rabbit polyclonal antibody for Exp#1 and Exp#2, respectively) for 2 hr. After washing three times, the cells were incubated with secondary antibodies (antirabbit Alexa647 and anti-mouse Alexa488, Invitrogen) for 1 hr, washed three times, and stained for DNA content using 405cellcycle (Invitrogen) and RNaseA for 30 min. Experiment in *Drosophila S2* cells was performed similarly, but only using the antibodies of Exp#2 (CellSignaling #9116 antibody used in Exp#1 does not recognize *Drosophila* Cdk1). Flow cytometry was performed using MACSQuant Analyzer (Miltenyi Biotec, U2OS cells) and FACSAria (Beckton Dickinson, S2 cells), and data analyzed using Bioconductor package flowCore. To assess the amount of unphosphorylated Cdk1 in cells in the G2 phase, G2 cells were gated using DNA content (blue channel), after which normalized Cdk1-P signal (red or green channel in Exp#1 or Exp#2, respectively) of was subtracted from normalized Cdk1 signal (green or red channel in Exp#1 or Exp#2, respectively) for each cell separately. U2OS and S2 cells in G2 phase were binned to 12 and 8 bins based on cell size (FSC-A), respectively, and the average and standard error calculated. *P* values for the bins were determined using Kolmogorov-Smirnov test against a center bin (7 and 4 for U2OS and S2, respectively).

### Microarray data analysis

RNA samples were obtained in several batches; all samples in a given batch were hybridized on Affymetrix *Drosophila* Genome 2.0 arrays at the same time using standard Affymetrix one cycle labeling and hybridization protocol. The raw CEL data were first reannotated according to custom CDF 12.1.0 (ENSG) described in Dai *et al.* [(Dai *et al.* 2005) http://brainarray.mbni.med.umich.edu/Brainarray/Database/CustomCDF/CDF_download.asp], and the expression values of the data corrected for background noise using RMA (Robust Multiple-array Average) normalization (Irizarry *et al.* 2003) using the Bioconductor R package. Differential expression from each experiment in each batch was assessed using a Bayesian linear model [R, LIMMA package, version 3.2.2 (Smyth 2004)]. For clustering analysis, the background corrected expression data were subjected to quantile normalization. Then, the average expression values of each experiment were subtracted from the average GFP values of their corresponding control experiments to obtain a log-fold difference value. Using LIMMA, we generated a shortlist consisting of 2792 significant genes in at least five experiments (absolute log fold change > 0.5, adjusted *P* value < 0.01). The dendrograms shown in Figure 2 and Figure S1 were generated using COSA distance clustering (Version 2, scaling factor λ = 0.6; http://www-stat.stanford.edu/∼jhf/COSA.html). Other packages used in the analyses were GOstats (v 1.7.4) and Drosophila2.db for GO overrepresentation analyses, and proxy (0.4-5) for Euclidean and Manhattan distance measurements. Correlation between gene expression and cell-cycle phenotypes was analyzed from log-transformed data using Spearman correlation. Because G1 decrease is commonly associated with abnormal cell-cycle phenotypes (death or polyploidy), the correlation coefficient was calculated from the samples in which G1 was increased. Significance was assessed by permutating the cell cycle phenotypes by batch, thus establishing a *P*-value cutoff that is expected to result in less than one correlated gene being identified by random for each phenotype. Scripts are available upon request. The original data and annotations were submitted to ArrayExpress in MIAME compliant form and are available (E-MTAB-453).

### Analysis of target gene overlap and network representations

For the percentage of overlap calculations in Figure S3B, experiments were selected with 50 or more significantly regulated genes (absolute log fold change > 0.3 at adjusted *P* value < 0.01; Benjamini and Hochberg). The significantly regulated gene lists of each of these experiments were then matched to the lists of all other experiments in this analysis and the percentage of overlap calculated in both directions. These percentages were then hierarchically clustered based on cosine angle distance metric. In the analysis of target gene overlap in Figure S4 and Figure S5A, we used an approach that is similar to that described in Goh *et al.* 2007, except that in our analysis the edges can be either positive (same effect on same genes) or negative (opposite effect on same genes), and their weight is not based directly on number of common target genes but on the overlap above that expected by random. To summarize in brief, for each experiment the significantly regulated genes were listed (over 0.3 absolute log fold regulated at *P* < 0.01) and then those genes were selected that were regulated in at least five experiments. Each experiment was paired to all other experiments, and the overlap expected by random was then compared with the observed overlap in both opposite and the same direction. The direction displaying larger overlap was taken, and the obtained overlap value normalized between 0 (less than or equal to random overlap) and 1 (complete overlap; scripts available on request).

The networks were constructed using in-house Perl scripts. Nodes with fewer than five adjacent edges were excluded from the networks (Figure 3, Figure 4, and Figure S6) except for the target overlap network (Figure S5A) from which only nodes without any adjacent edges where excluded. Networks were uploaded into Cytoscape network visualization software (Shannon *et al.* 2003) in xgmml format. Network layouts were generated using yFiles organic algorithm, which is one variant of the force-directed layout paradigm intended to show the clustered structure of the graph.

### Chromatin immunoprecipitation-sequencing (ChIP-seq)

ChIP-seq was performed essentially as described in (Tuupanen *et al.* 2009) with the following modifications.

#### ChIP for Myc and Max:

S2 cells were formaldehyde crosslinked, sonicated, and immunoprecipitated with the following set of antibodies (Santa Cruz Biotechnology, Inc.): Myc: dN-20 and d1-717, sc-15832 and sc-28207. Max: d1-160, sc-28209. IgG controls sc-2027 and sc-2028.

#### ChIP for E2f and Myb:

S2 cells were transiently transfected with plasmids (backbone pRmHa-1) containing metallothionein promoter driven Cterminally His-streptavidin binding peptide-3xV5 tagged Drosophila open reading frames. Expression of the genes was induced 24 hr after transfection by 0.5 mM CuSO_4_, and the incubation continued for 48 hr until formaldehyde crosslinking was done. Immunoprecipitations were made with 7.5 μg of monoclonal anti-V5 antibody (46-1157; Invitrogen) or IgG control antibody (sc-2025; Santa Cruz Biotechnology, Inc.).

Library preparations were done by repairing the immunoprecipitated DNA using Klenow and T4 DNA polymerase and T4 polynucleotide kinase (MBI Fermentas, Vilnius, Latvia), and ligating the Illumina sequencing adapters according to manufacturer’s instructions. Subsequently, PCR-amplified fragments of approximately 180−350 bp were sequenced using Illumina Genome Analyzer. Complexity of the sequencing libraries was estimated, fragments mapped to the genome, and peaks called as described in Wei *et al.* (2008). Genes that contained a significant (*P* < 0.01) peak within 2 kb of their transcription start sites were then identified and used in the validation of the microarray data.

### Gain of function fluorescence-activated cell sorting (FACS)

For the gene overexpression analysis 0.25 × 10^6^ S2 cells were seeded into 24-well format ∼16 hr before transfection. Cells were then transfected with 1.5 μg of plasmid DNA (gene open reading frame fused with C terminal AGT-2xproteinG tag under the control of opIE2 promoter) together with 5 μL of Fugene HD. After 72 hr, the cells were washed once with [1% BSA in Tris-buffered saline (TBS)] and fixed for 48 hr with ice-cold 70% ethanol. Fixed cells were washed three times (1% BSA in TBS) followed by overnight incubation with Alexa Fluor 488goat anti-mouse IgG (Invitrogen). Next day cells were washed four times (1% BSA in TBS) and incubated overnight with Draq5 DNA-dye (Cell Signaling Technology). Flow cytometry analysis was performed using a FACSArray flow cytometer (Beckton Dickinson). Flow cytometry graphs were then analyzed using FACSDiva software (Beckton Dickinson, FACSArray). The separation between positive and negative cells was adjusted with nonexpressing ORFs.

## Results

### Identification of target genes using RNAi followed by expression profiling

**Figure 1:**
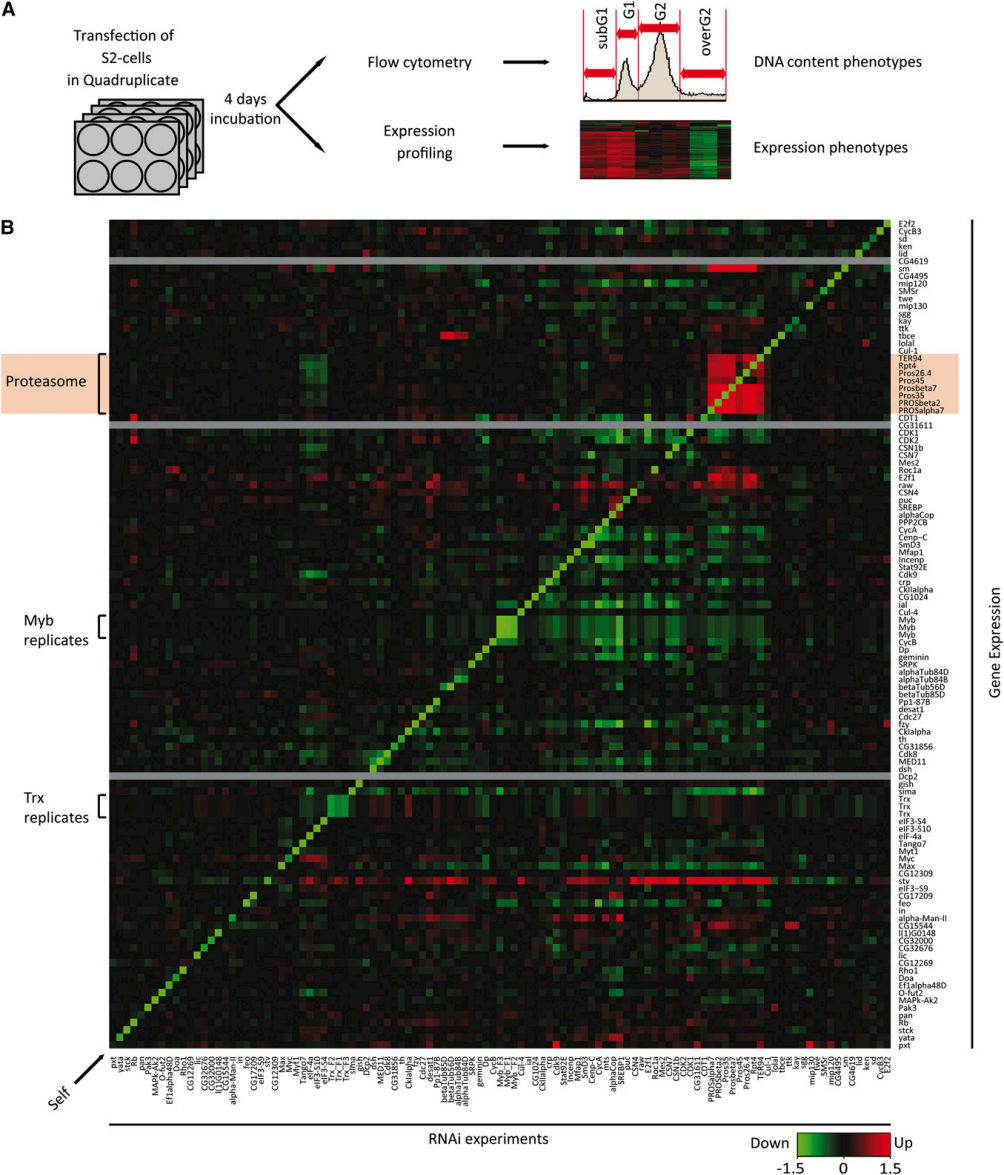
Targeting cell cycle regulators by RNAi. (A) Schematic representation of the experimental setup. (B) Effect of RNAi-mediated knockdown (RNAi experiments; columns) of 107 cell-cycle regulatory genes on the expression of the same set of genes (rows). The three genes not present on the array are indicated by gray bars. Samples and genes were ordered on the basis of similarity of the effect of the RNAi treatment on the expression of the target genes shown (hierarchical clustering, Euclidian distance metric). Inset indicates the color scale (log_2_ fold change of gene expression compared with GFP RNAi control, green represents decrease and red increase). Note that the vast majority of the RNAi treatments strongly decrease expression of the intended target gene (Self, arrow), and replicate samples targeting different regions of the same genes (*Myb*, *Trx*, constructs F1, F2, and F3) cluster together and have similar effects on gene expression. Note also that RNAi-induced decrease in expression of all analyzed components of the proteasome result in increase in expression of all the other proteasome components (red box with green diagonal line, gene names on pink background). For some genes human ortholog names are used, see Table S11 for details.

Despite the central importance of cell growth and division in developmental biology and cancer, the transcriptional networks that control these processes have not been mapped in a genome-wide scale. To gain systematic understanding of the transcriptional control of cell growth and cell cycle, we determined the effect on expression of all genes in cultured *Drosophila* S2 cells after individual loss of 23 transcription factors and 84 other genes with strong cell cycle or cell size phenotypes [(Bjorklund *et al.* 2006) see Table S1]. For each knockdown, double-stranded RNA-transfected cells of the same cultures were analyzed in parallel for DNA content and cell size using flow cytometry (FACS), and for steady-state mRNA levels using Affymetrix *Drosophila* genome 2.0 arrays (Figure 1A). We also analyzed the mRNA expression levels of these knockdowns using RNA seq to serve as internal controls of the Affymetrix results (Figure S1, Table S2).

**Figure 2:**
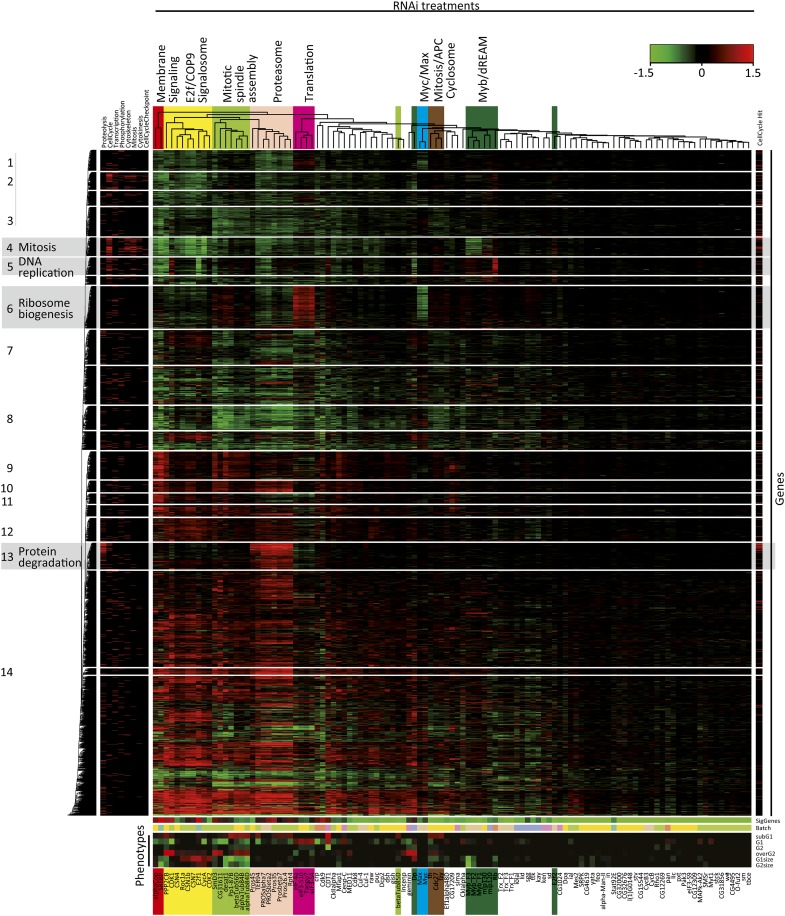
Two-dimensional clustering of the expression profiling data. Samples and target genes were clustered using the COSA algorithm (λ = 0.6, average linkage) based on expression of genes that were significantly regulated in five or more samples. Sample subclusters that contain multiple members of the same protein complex or signaling pathway are named according to the known function of the genes (top), and indicated by color. Heatmap below the dendrogram indicates the number of significant genes (top row; 0 genes, bright green; more than 1500 genes, red; see also Table S2), the batch in which the sample was processed (second row), and the cell-cycle phenotypes (bottom rows of heatmap). Note that samples with similar cell-cycle phenotypes cluster together even when the clustering is based solely on gene expression, and that sample batch does not strongly influence clustering of samples in which moderate to high number of genes are regulated. The gene clustering analysis revealed multiple sets of genes that clustered together based on the similarity of their expression across the samples. Fourteen such clusters (indicated by numbers on the left) were selected for further analysis. Red lines in left and right sidebars indicate GO terms and cell cycle or cell size regulators (Bjorklund *et al.* 2006), respectively. Gene clusters enriched in the GO terms mitosis, DNA replication, ribosome biogenesis, and the protein degradation are indicated by a gray background.

In a large majority of the experiments (89%), the targeted gene was among the top 1% of down-regulated genes (Figure 1B, Figure S2, Table S3). In addition, targeting genes with similar biological functions (*e.g.*, proteasome components), or targeting the same gene with multiple nonoverlapping dsRNA constructs resulted in similar expression profiles (Figure 2). Our results also were consistent with earlier data in the limited number of cases in which published array results using the same platform were available [see *Materials and Methods* and Table S4 (Reddy *et al.* 2010)].

### Clustering analysis of the samples

We next used the generated compendium of expression profiles to identify which of the knocked down genes act in pathways of the direct transcriptional regulators. This approach is similar to delineating signaling pathways using classical forward genetics, except that here the perturbations are known, the quantitative expression profile replaces the morphological phenotype, and formal computational methods replace the visual inspection (Holstege *et al.* 1998; Hughes *et al.* 2000). Potential pathways were then identified as groups of genes whose loss resulted in a similar or inverse expression profiles.

The similarity of expression profiles was assessed using hierarchical clustering (Hughes *et al.* 2000; Sherlock 2000) based on similarity scores determined by the COSA algorithm [v2; (Friedman and Meulman 2004)]. Clustering analysis of the expression profiles yielded very similar patterns for both the array hybridization and the RNA seq, indicating that the analysis was robust to the method used to generate the expression profiles (Figure 2, Figure S1). Furthermore, the experiments in the expression profiling clusters also often showed similar cell cycle FACS profiles, confirming that the expression dataset is relevant to the regulation of the cell cycle (Figure 2, bottom). Targeting genes that participate in similar cellular processes such as *Myc* and *Max* (Gallant *et al.* 1996) yielded expression profiles that clustered together (Figure 2). Other easily identified clusters in Figure 2 are genes of the Proteasome (*Pros35*, *pros45*, *Prosalpha7*, *Prosbeta2*, *Prosbeta4*, *Pros26.4*, and *Rpt4*), the dREAM complex (*Myb*, *mip120*, and *mip130*), and translation initiation factors (*eIF-4a*, *eIF3-S10*, and *eIF3-S4*). Interestingly, the translation cluster also contained the gene *Tango7*, which has previously been linked to a very large number of RNAi phenotypes (Boutros *et al.* 2004; Friedman and Meulman 2004; Bard *et al.* 2006; Bjorklund *et al.* 2006; Beller *et al.* 2008; Guo *et al.* 2008; Chew *et al.* 2009; Doumanis *et al.* 2009). Here we also found a potential role for the COP9 signalosome in regulation of the Cdk/E2f pathway in S2 cells as the expression profiles of *Cdk1*, *Cdk2*, *CyclinA*, and *E2f* clustered together with those of components of the COP9 signalosome (*Csn1b*, *Csn4*, and *Csn7*).

### Analysis of target gene overlap

Hierarchical clustering imposes a tree-like structure on the data, even if the underlying structure is better represented by a network. It thus commonly fails to identify similarity between a treatment that affects a number of target genes, and other treatments that affect mutually exclusive subsets of this same set of genes. Such cases can be biologically relevant and to identify these, we used several methods to determine the overlap of the significantly regulated target genes between all samples (Figure S3B, Figure S4, Figure S5A). For clarity, we refer to all genes regulated in response to loss of a gene as targets of that gene, irrespective of whether the effect is direct or indirect.

For each pair of expression analyses, we calculated the excess gene overlap over that expected by random (see *Materials and Methods* for details). We then used a deterministic force-directed layout algorithm (y-files organic) to generate a network representation of the matrix, where nodes—representing RNAi treatments—repel each other unless they are connected by an edge representing overlap in their target gene sets.

Using this approach, we classified several interactions of samples that displayed partial overlap with two or more other samples that did not necessarily have common target genes. For example, Mip130 targets overlapped with those of Myb and E2f2, whereas Myb and E2f2 targets did not overlap with each other (Figure S3B, Figure S4, Figure S5A; File S2). This finding suggests that *Drosophila* Mip130 participates in two separate complexes, one containing E2f2 and the other Myb. The presence of Mip130 in distinct E2f2 and Myb containing complexes is consistent with what has been observed in human cells (Pilkinton *et al.* 2007) but contrary to previous reports that claimed that Myb-MuvB/dREAM in *Drosophila* contains both E2f2 and Myb (Korenjak *et al.* 2004; Lewis *et al.* 2004; Lipsick 2004). Further analysis at the protein level by tandem-affinity purification followed by mass spectrometry supports our expression data on the separation of the E2f2 and Myb complexes in *Drosophila* (Turunen *et al.*, in preparation 2012). The overlap analysis (Figure S3B, Table S5) also indicated that there is cell-cycle regulation control at the lipid level. Two proteins linked to membrane trafficking and synthesis, coatomer I (*alphaCop*) and *Drosophila* stearoyl-CoA 9-desaturase (*Desat1*), regulate the cell cycle via the transcription factor Sterol regulatory element binding protein [*SREBP*/*HLH106* (Bjorklund *et al.* 2006)].

In addition, the overlap analysis allows the identification of interactions between samples that affect a relatively small number of target genes and which thus get “buried” in the hierarchical clustering analysis. For example, we found that knockdown of *Lolal* and the C2H2 Zinc finger protein *Ken* resulted in overlapping expression profiles (Figure S3B, Figure S4, Figure S5A). This finding is supported by data from the high-throughput yeast two-hybrid screen of Giot *et al.* (2003). We were also able to validate this interaction by using tandem-affinity purification followed by mass spectrometry and yeast two-hybrid analysis (Figure S5B and not shown).

### Analysis of the target genes

To further assess the relevance of this dataset to the cell cycle, we combined all target gene data into a network (Figure S6, File S2), which revealed that the target gene sets of the RNAi treatments were highly interconnected. More than 4.9 × 10^5^ instances of a shared gene target were observed between RNAi treatments, compared with 8.0 × 10^4^ expected by random. Furthermore, many target gene sets were enriched in genes belonging to the same gene ontology (GO) categories; the GO terms “chromosome segregation,” “mitotic cell cycle,” “cell cycle,” and “chromosome organization” were among the ten most commonly overrepresented, with at least one of these terms being found overrepresented (*P* < 0.001) in 17 of the 107 target gene sets (Table S6).

For 20 of the dsRNAs knockdowns, the target gene sets were significantly enriched with genes whose loss affects the cell cycle as identified by Bjorklund *et al.* 2006 (*P* < 0.01, hypergeometric test; Table S6). On the array, 541 genes of these genes were represented and 392 of them were significantly regulated in at least one sample (*P* < 0.001). The major transcription factor controlling the cell cycle, E2f, and the most important Cdk, Cdk1 (Santamaria *et al.* 2007; Coudreuse and Nurse 2011), were located at the center of the network describing regulation of identified cell cycle genes by the RNAi treatments (Figure S6). Taken together, these data show that an unbiased RNAi screen of the whole genome, followed by whole-genome transcriptional analysis of genes identified in the screen allows discovery of central aspects of the cell cycle regulatory network.

### Clustering of target genes based on coexpression across RNAi treatments

Of special interest within the compendium of expression profiles are genes that are coregulated across all or a subset of the RNAi treatments. Analysis of such genes should be particularly powerful in identifying genes that control cyclical processes. For example, RNAi treatments causing cell-cycle arrest at either G1 or G2 would be expected to result in loss of expression of genes involved in S-phase of the cell cycle (*e.g.*, DNA replication). Two-dimensional clustering using the COSA method resulted in identification of 14 clear clusters of genes that were coexpressed across RNAi treatments (Figure 2, boxes). To see whether these clusters contained biologically relevant data concerning the cell cycle, we analyzed the associated GO terms of the gene sets contained in the identified clusters and checked whether any of them were significantly overrepresented. This revealed that clusters 4, 5, 6, and 13 were highly enriched in genes linked to mitosis (M; *P* < 3.8 × 10^−30^), DNA replication (S; *P* < 2.1 × 10^−49^), ribosome biogenesis (*P* < 2.3 × 10^−25^), and protein degradation (*P* < 7.6 × 10^−25^), respectively (Figure 2, clusters in gray background; Table S7). Thus, knockdown of genes that affect the cell cycle followed by clustering of genes based on coexpression very effectively classified genes according to biological subprocesses that are central to regulation of the cell cycle.

**Figure 3:**
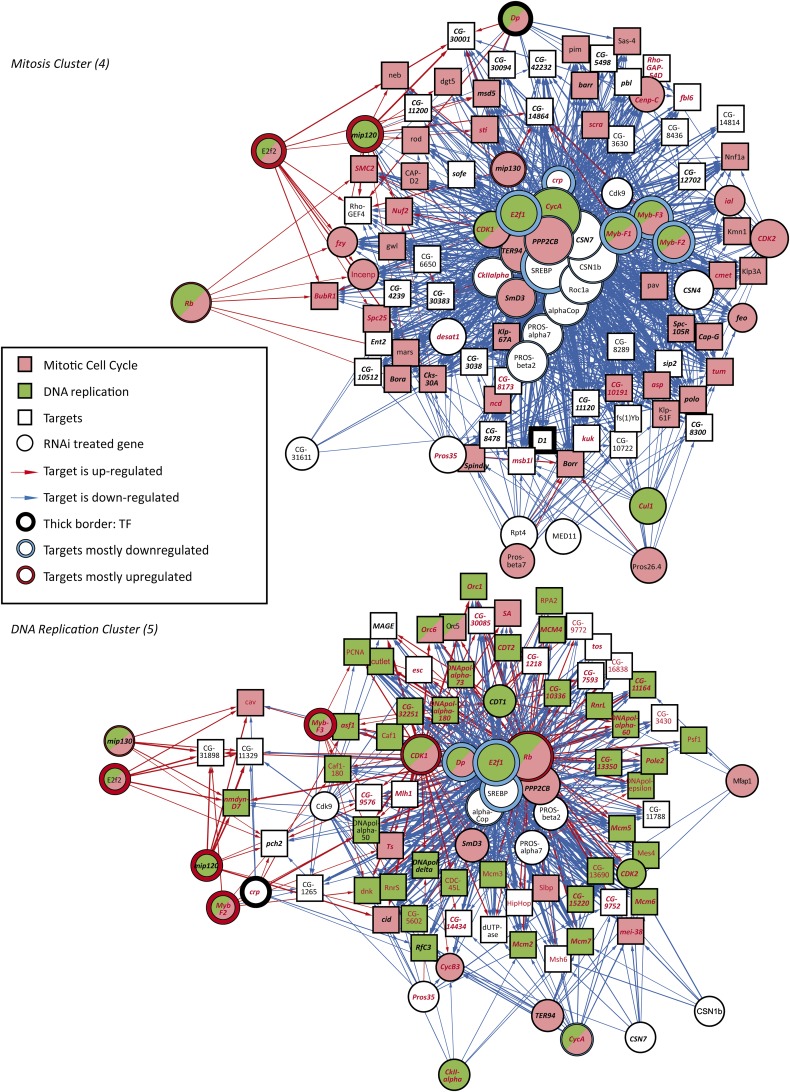
Subnetworks regulating mitosis (top) and DNA replication (bottom). Nodes are connected by an edge if an RNAi-treated gene (circle) results in regulation of a target gene (box). Thickness of the edge represents the magnitude of the effect, and its color indicates up-regulation (red) or down-regulation (blue) of a target gene after RNAi. Size of the circles indicates the number of target genes regulated. Nodes are colored according to GO annotations indicated in the inset (see Table S12 for details). Only target genes that show similar gene expression across the samples (clusters from Figure 2) are included in the subnetworks. Target genes from ChIP-seq experiments are also indicated. Key: E2f (red typeface), Myb (bold italic). Note that Myb, SREBP, E2f, and Crp transcription factors, (thick borders) are located at the center of the subnetwork of the mitosis cluster.

**Figure 4:**
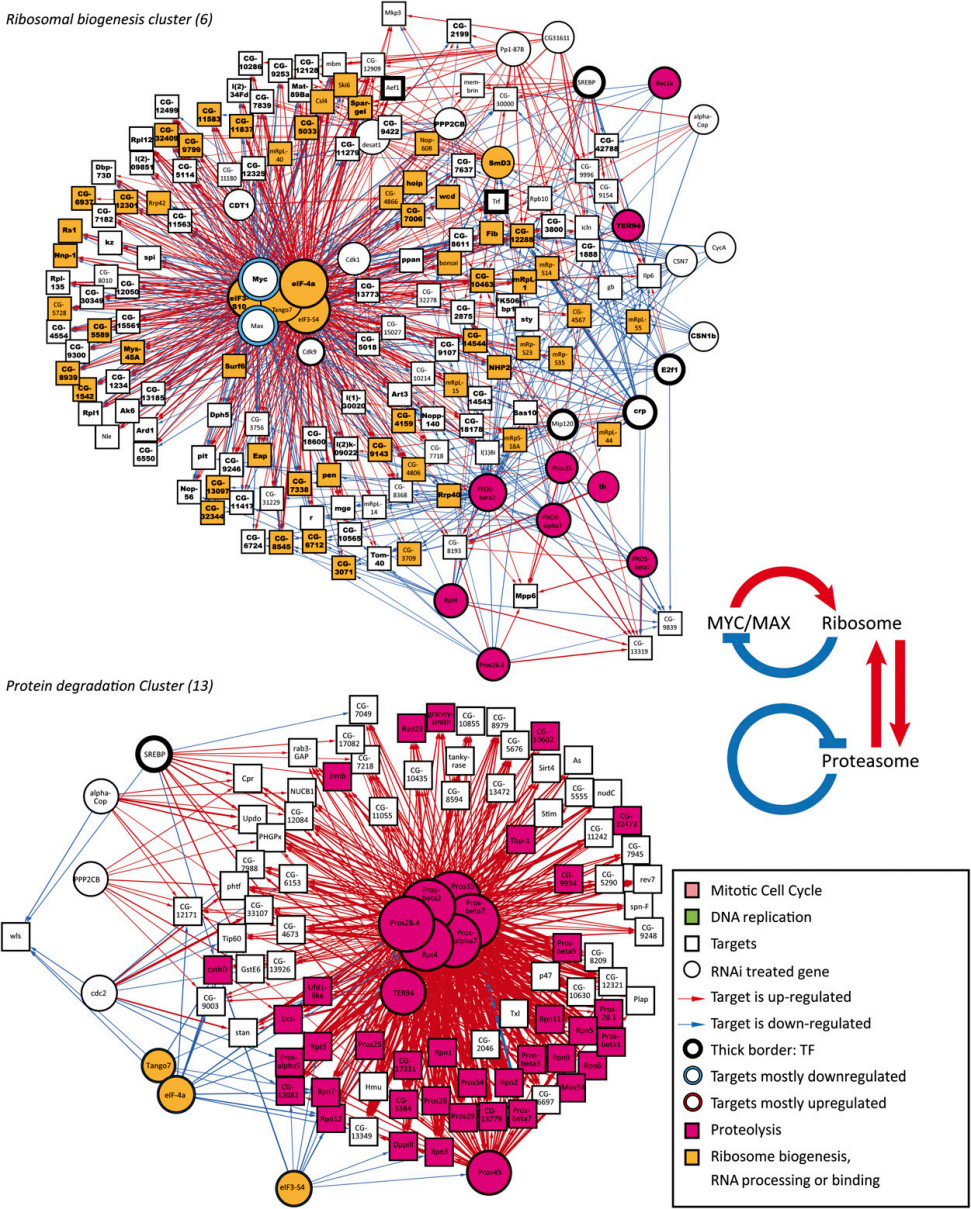
Subnetworks regulating ribosome biogenesis (top) and protein degradation (bottom). Nodes are connected by an edge if an RNAi-treated gene (circle) results in regulation of a target gene (box). Thickness of the edge represents the magnitude of the effect, and its color indicates up-regulation (red) or down-regulation (blue) of a target gene after RNAi. Size of the circles indicates the number of target genes regulated. Nodes are colored according to GO annotations indicated in the inset (see Table S9 for details). Only target genes that show similar gene expression across the samples (clusters from Figure 2) are included in the subnetworks. Also, knockdown of the proteasome subunits results in strong up-regulation of a large number of proteasome components (red) and loss of translation initiation factors results in downregulation of a subset of proteasome subunits (yellow, bottom-left). Target genes from ChIP-seq experiments are also indicated. Key: Myc and Max (bold). Note that *Drosophila* Myc/Max and translational regulators localize to the center of network in the top panel and target the same genes involved in ribosomal biogenesis, but with opposite effects. Inset shows the feedback between ribosome and Myc/Max, and the homeostatic feedback between ribosome and proteasome.

To further analyze these results and identify the main regulators of these gene sets, we generated network representations of the target-gene relationships within gene clusters 4, 5, 6, and 13, and laid them out using the deterministic force-directed layout algorithm described previously. This unsupervised analysis identified the genes *E2f*, *Dp*, and *Rb* as key regulators of DNA replication (Figure 3, bottom; File S4). For the mitosis cluster, the genes coding for the central transcription factors are *SREBP*, *E2f*, *Myb*, and *cropped* (*crp*; Figure 3, top; File S3). This cluster consists almost exclusively of genes important for mitosis and/or cytokinesis. The ribosome biogenesis cluster in turn is heavily enriched in genes that are strongly up-regulated by loss of translation initiation factors (Figure 4, top; File S5). This feedback response appears to be mediated by the Myc/Max transcription factor because *Myc* expression is strongly induced by loss of the translation initiation factors (Table S2), and the same set of target genes is down-regulated by loss of expression of either *Myc* or *Max* (Figure 4, top). A subset of the genes in the ribosome biogenesis cluster were also down-regulated by loss of the proteasome (purple), indicating that there is feedback between Myc/Max, the ribosome, and the proteasome (Figure 4, inset). Interestingly, the target genes in the protein degradation cluster were also down-regulated by loss of the translation initiation factors and strongly up-regulated by loss of the proteasome (Figure 4, bottom; File S6). Taken together, the expression data from clusters shown in Figure 4 indicates that a complex homeostatic circuit exists to balance cellular protein synthetic and catabolic capacity (Figure 4, inset).

### Validation of target genes using ChIP-seq

To examine the identified target gene relationships and establish which interactions were direct or indirect, we performed ChIP-seq experiments for the transcription factors E2f, Dp, Myb, Myc, and Max that, according to the expression data, targeted many of the genes in DNA replication, mitosis and ribosome biogenesis clusters, respectively.

Genes showing a significant peak within 2 kb of their transcription start sites were classified as likely to be direct targets of the respective transcription factors (ChIP-seq targets, see *Materials and Methods* for details). In the DNA-replication cluster, the majority of the genes were E2f ChIP-seq targets (peak enrichment *P* < 2.7 × 10^−33^, hypergeometric test). In the mitosis cluster, both ChIP-seq targets of Myb and E2f were overrepresented (1.1 × 10^−12^ and 6.0 × 10^−6^, respectively). The ribosome biogenesis cluster, in turn, showed strong enrichment in Myc and Max ChIP-seq targets (*P* < 5.7 × 10^−70^). Thus, in each case, we were able to validate the major transcription factor regulating the clusters using an independent method (Figures 3 and 4).

### Correlation between expression profiling and cell-cycle phenotypes

**Figure 5:**
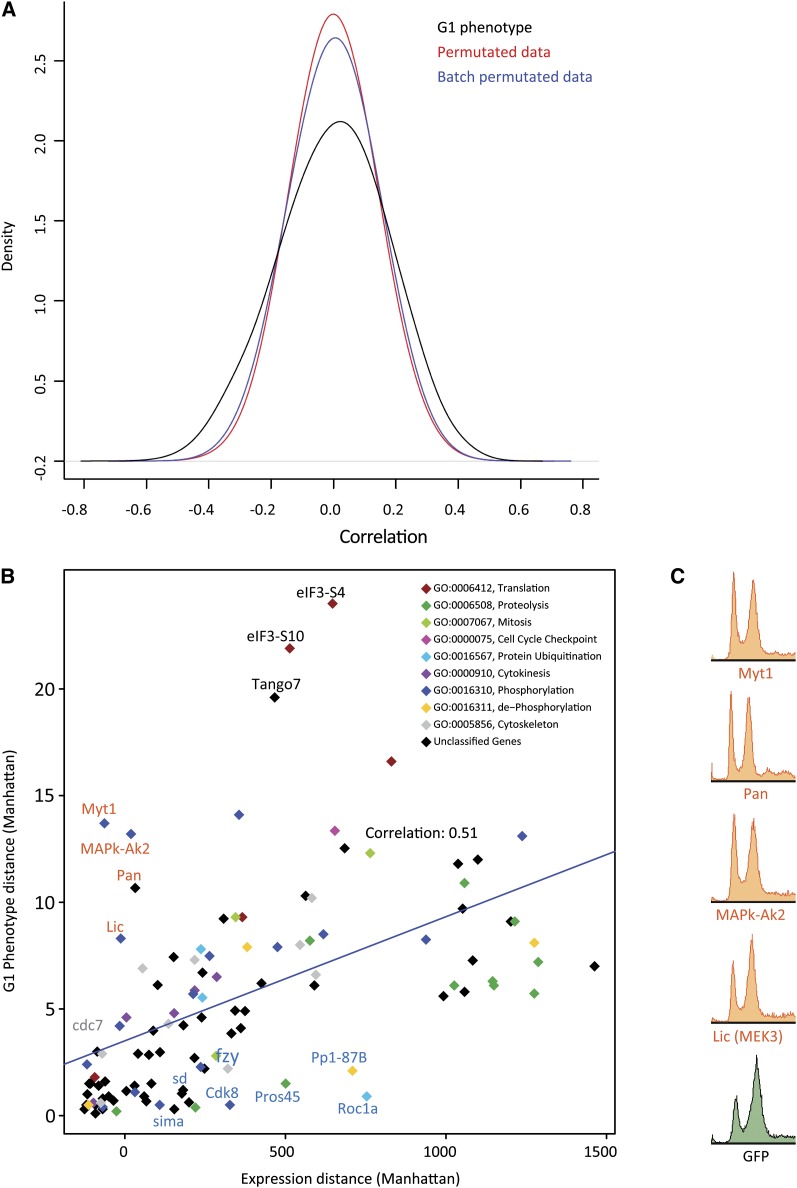
Correlation between gene expression and G1 content. (A) Density plot of correlation coefficients between gene expressions and G1 content (black line) shows that expression of a subset genes correlate with G1 phenotype better than expected by random. Colored lines represent randomized data, permutated globally (red) or according to treatment batch (blue). Note that maximum correlation is relatively weak (less than 0.6 in either direction). (B) G1 phenotype strength plotted as a function of total effect (Manhattan distance) of the same RNAi treatment on gene expression (effect of GFP dsRNA normalized to 0). Although there is overall correlation between G1 and gene expression, several genes have much stronger effect on either G1 (red typeface) or gene expression (blue typeface). (C) Flow cytometric phenotypes of the four experiments showing a strong G1 phenotype with little effect on gene expression.

The main benefit of this dataset is that it directly links expression profiles to specific cell-cycle phenotypes. Therefore, we looked for correlation between gene expression and cell-cycle FACS phenotype. First, we identified individual genes whose expression correlated with the cell cycle phenotypes across all the RNAi treatments. This analysis revealed that although the expression of some genes does correlate with phenotype, the correlation was relatively weak (Figure 5A, Table S8). For the identified genes with the greatest correlation coefficients with G1 phenotype, we did not find enriched associated GO categories (see *Materials and Methods* for details). Genes whose expression correlated with G2 were weakly enriched for GO annotations involved in spindle assembly. Strongest enrichment of GO categories was observed for genes whose expression correlated with cell size at G1; these were enriched in cell cycle regulators, and RNA polymerase II complex components and adaptor proteins [Table S9; see also (Bjorklund *et al.* 2006)].

Next, to have a more global view of the correlation between gene expression and the cell cycle, we plotted for each RNAi treatment the change in G1 phenotype—which displays the largest shifts—as a function of the total magnitude of change in expression level of all genes that were used in the clustering analysis. This analysis revealed that there was a general correlation between the cell cycle and gene expression phenotypes (r = 0.51; Figure 5B). However, some RNAi treatments stood out clearly and resulted in very strong G1 increase FACS phenotypes while having a very limited effect on gene expression (Figure 5, B and C), effectively uncoupling transcription from cell-cycle progression. Knockdown of expression of these genes—*Myt1*, *MAPk-Ak2*, *licorne*/*MEK3*, and *Pangolin*—significantly affected only 1, 2, 6, and 64 genes, respectively, and none of these target genes was shared between all of them (Figure S7A). Interestingly, we found that all of these genes directly or indirectly regulate mitosis via modulation of Cdk1 activity. The kinase Myt1 negatively regulates Cdk1 by phosphorylation at threonine 14 and tyrosine 15 (Edgar and O’Farrell *et al.* 1989, 1990; Mueller *et al.* 1995; Jin *et al.* 2008), and the genes *licorne/MEK3* and *MAPk-Ak2* belong to the p38β mitogen-activated protein kinase pathway that has been reported to negatively control the phosphatase string/Cdc25, which dephosphorylates these Cdk1 residues (Manke *et al.* 2005). Our results also show that the Wnt pathway regulated transcription factor Pangolin (pan; Table S2) strongly represses *CDC25/string* in S2 cells—*string* is the third-highest up-regulated gene in response to loss of pan. Conversely, knockdown of *string* expression through RNAi results in larger cells that arrest in G2 and show DNA re-replication (data not shown), a phenotype that closely resembles that of *Cdk1* RNAi−treated cells. Indeed, transcriptional analysis of cells treated with String dsRNA revealed that more than 64% of the significantly affected genes are also significantly affected in Cdk1 knockdown experiments.

A potential role for the uncoupling of transcription and cell cycle progression would be in controlling cell size because both large and small cells need similar amounts of DNA-replication proteins and nucleotides for cell division. This hypothesis predicts that (1) increased Cdk1 activity would be associated with decreased cell size; (2) down-regulation of the genes that showed strong G1 increase phenotype and weak effect on gene expression would result in decreased cell size; and (3) Cdk1 phosphorylation would be a function of cell size.

**Figure 6:**
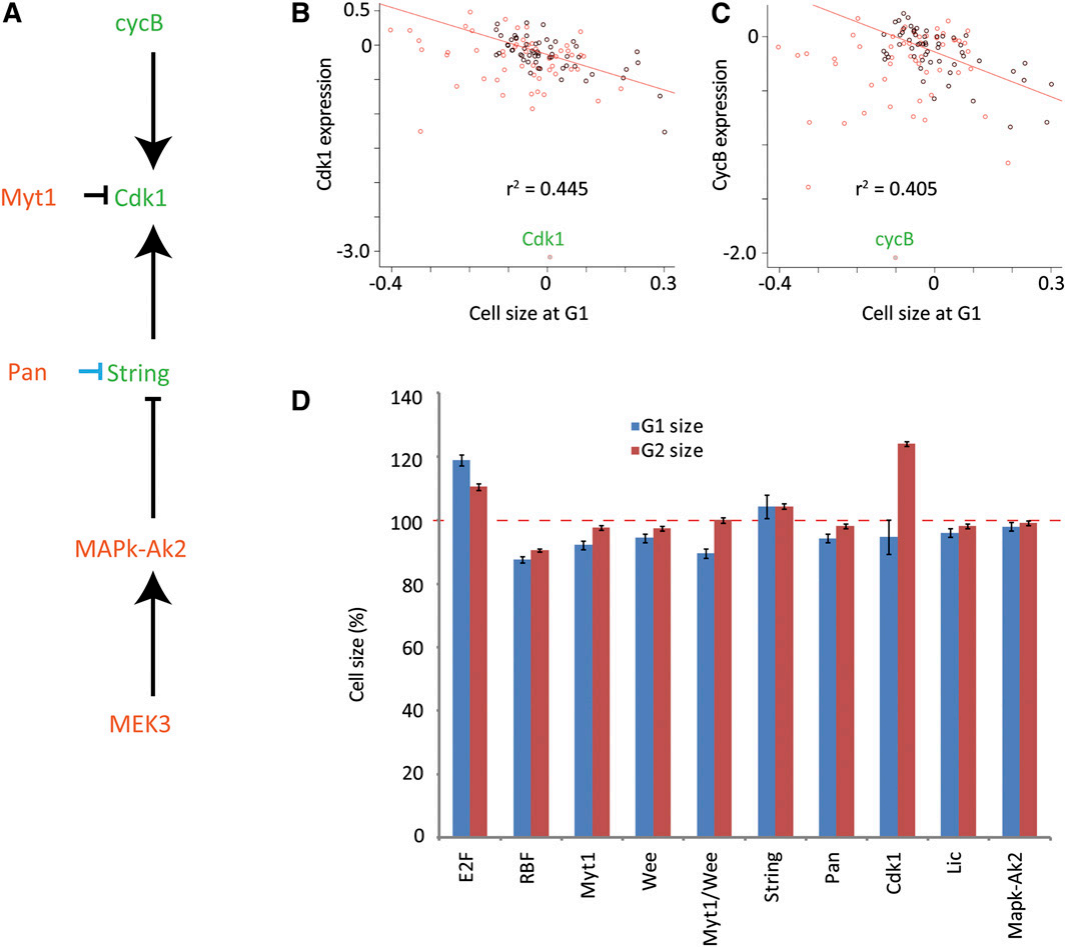
Cdk1 activity is associated with cell size. (A) All genes that increase G1 and have little effect on gene expression are regulating the *Cdk1* pathway. Blue indicates regulation at the expression level, black at the protein level. (B and C) Correlation between cell size and expression of *Cdk1* and its activator *cyclinB*. Samples where G1 size was increased or decreased are in black or red, respectively. As G1 decrease was commonly associated with abnormal cell cycle phenotypes (death or polyploidy), the correlation coefficient was calculated from the G1 increased samples using a linear model. (D) Cell size is affected by RNAi of cell-cycle regulators. Percent increase/decrease of the cell size in G1 (blue) and G2, (red) is plotted for cells treated with the indicated RNAi constructs compared to the GFP control experiments. Error bars indicate the margin of error at 95% confidence.

In metazoans, Cdk1 activity is primarily controlled by three mechanisms, mRNA expression, binding of B-type cyclins, and inhibitory phosphorylation (Lee *et al.* 1988; Lehner and O’Farrell 1990). We first analyzed correlation of *Cdk1* and *CyclinB* mRNA expression with cell size in G1. The *Cdk1* and *CyclinB* genes were among those that showed the strongest negative correlation with G1 cell size (Figure 6, B and C), indicating that increased *Cdk1* and *CyclinB* mRNA levels are associated with decreased cell size (*P* < 3.3 × 10^−6^ and 2.0 × 10^−5^, respectively). The effect of *Cdk1* expression levels on cell size was causative and not merely correlative, as indicated by the fact that targeting of *Cdk1* by RNAi also resulted in increased G2 cell size (Figure 6D; *string* and *Cdk1* knockdown arrests cells strongly in G2, and almost no cells remain in G1). Furthermore, analysis of the G1 and G2 cell-size phenotypes revealed that all treatments affecting G1 content without effect on gene expression also decreased average cell size (Figure 6D).

**Figure 7:**
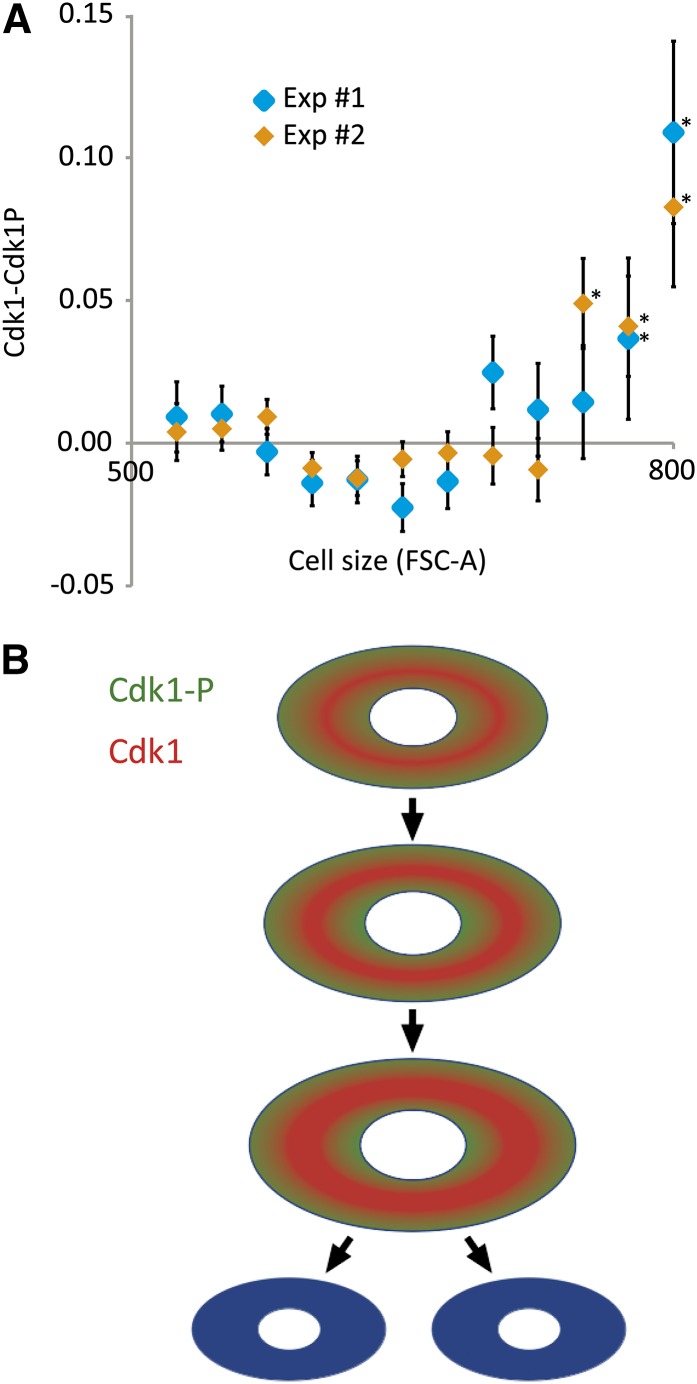
Regulation of cell size by Cdk1. (A) Relative amount of unphosphorylated Cdk1 (Cdk1-Cdk1P) in human U2OS osteosarcoma cells as a function of cell size (FSC-A). Two experiments using different sets of antibodies are shown. Note that unphosphorylated Cdk1 rapidly increases after the G2 cells reach a certain size. This non-linear increase is consistent with the fact that the Cdk1 inhibitory kinases Myt1 and Wee1 are localized in membranes and nucleus, respectively, whereas Cdk1 is soluble. Error bars indicate one standard error, and asterisks indicate *P* < 0.01 (Kolmogorov-Smirnov test). (B) Model of regulation of cell size. Different cells in a multicellular organism vary greatly in size, but sizes within a cell type are more constant. We propose that the constant cell size is maintained by the following mechanism: In small cells, Myt1 and Wee1 phosphorylate Cdk1 and convert it to Cdk1-P (green). When cells become larger, membrane bound Myt1 and nuclear Wee1 are unable to efficiently phosphorylate Cdk1, leading to cell size-dependent increase in Cdk1 activity (red) and entry into mitosis. In this model, setting the target size for different cell types requires an additional mechanism that regulates the concentrations of the soluble reactants (Cdk1, B-type cyclins and the string/Cdc25 phosphatase). Regulation of mRNA expression of both *string/Cdc25* (Lehman *et al.* 1999; Grosshans and Wieschaus 2000) and *Cdk1* (Lee *et al.* 1988; Lehner and O’Farrell 1990) has been described, and we show here that mRNA levels of *Cdk1* and *cyclinB* correlate with cell size (see Figure 6, B and C).

Finally, we tested whether Cdk1 phosphorylation is a function of cell size. For this, we analyzed cell size together with levels of both total Cdk1 and phosphorylated Cdk1 in the same cells by using flow cytometry. To eliminate artifacts, such experiments are best performed by using two different pairs of antibodies because this controls for antibody cross-reactivity and also allows switching of the fluorescence channels. We were unable to find two sets of antibodies binding to phosphorylated and unphosphorylated *Drosophila* Cdk1; therefore, we also performed these experiments in the commonly used cell-cycle model, human U2OS cells. We found that consistently with the hypothesis, the amount of unphosphorylated Cdk1 increases as a function of cell size in the G2 phase of the cell cycle in both human and *Drosophila* (Figure 7A, Figure S7B). The results in *Drosophila* S2 cells were found with the single antibody pair available (Figure S7B). Altogether, these results confirm our hypothesis and suggest that regulation of Cdk1 activity contributes to a mechanism that regulates cell size in animal cells.

## Discussion

Our results represent the first genome-scale loss-of-function study of a transcriptional network controlling cell division in any metazoan organism. We show that cellular pathways can be identified by simple clustering of the expression profiles and that a combination of these data with cell-cycle phenotypes allows the identification of genes specific for the regulation of different stages of the cell cycle. The experiments were conducted in batches under similar growth conditions, and each batch had its own control experiment as a reference. The inclusion of controls into each batch allowed us to largely eliminate batch variation, permitting direct comparison of the expression profiles over different sets of experiments. The entire set of data consisting of more than 3.48 million individual measurements will be made available both as primary data in ArrayExpress (accession E-MTAB-453) and as machine readable tables and networks (File S1; File S2; File S3; File S4; File S5; File S6; File S7), providing a rich resource for analysis of individual interactions that regulate cell cycle processes.

### Network controlling growth and protein homeostasis

By clustering of the expression profiles, we identified several processes that control the cell cycle, some of which have been previously described, and some of which were unknown. Many genes that are required for cell-cycle progression are components of three large and ancient macromolecular machines, the ribosome, the proteasome and the COP9 signalosome (Wei *et al.* 2008; Besche *et al.* 2009; Frank and Gonzalez 2010). Of these, we find that the ribosome and proteasome are regulated at the level of gene expression and form a positive feedback loop that maintains overall protein homeostasis. That is, loss of protein synthetic capacity results in decrease of proteasome mRNA expression, and conversely loss of proteasome activity results in a decrease of mRNAs for genes involved in ribosome biogenesis. Such process-level feedback has been described earlier in yeast (Xie and Varshavsky 2001; Jorgensen *et al.* 2004), but the functions of the transcription factors involved are not conserved in *Drosophila*.

Interestingly, profiles generated by RNAi targeting the eukaryotic initiation factors (*eIF-4a*, *eIF3-s10*, *CG8636*/*eIF3-S4*) are also highly similar to the profiles resulting from loss of either component of the dimeric transcription factor Myc/Max. This relation is inverse, that is, genes that are down-regulated by loss of Myc/Max are up-regulated by loss of the eIFs and vice versa. Loss of translational regulators increases *Myc* mRNA, and conversely loss of Myc or Max strongly down-regulates genes involved in ribosome biogenesis. Validation of this target gene set by ChIP-seq revealed that almost all of these genes are direct targets of both Myc and Max. Combined with the results of the proteasome subunits, this suggests that the highly specific feedback circuit involving Myc/Max connects to the protein synthesis/degradation network. This feedback loop would maintain cellular protein synthetic capacity, and also allows for external growth stimulatory pathways to act through Myc to control the protein synthetic activity of cells [see also (Grandori *et al.* 1996; Zaffran *et al.* 1998; Greasley *et al.* 2000; Xie and Varshavsky 2001; Grewal *et al.* 2005; Lundgren *et al.* 2005)]. The entire homeostatic circuit is of particular importance due to the central role of cellular protein homeostasis (proteostasis) in aging and disease (for review, see Balch *et al.* 2008; Rajalingam and Dikic 2011). Our results highlight the specificity of the Myc/Max−ribosome feedback circuit (Figure 4), and suggest that tumorigenesis due to loss of ribosomal subunits (Amsterdam *et al.* 2004; Narla and Ebert 2010) may result from aberrant feedback that increases Myc/Max activity. Interestingly, this system is also coupled to the cell cycle. Loss of translational activity leads to arrest at G1, whereas loss of the proteasome arrests cells at G2.

### Specificity of RNAi screens

dsRNAs targeting one gene that has previously not been linked to translation in *Drosophila*, *Tango7*, also resulted in expression profile that was very similar to that induced by loss of the translational initiation factors *eIF-4a*, *eIF3-S10*, and *CG8636/eIF3-S4* (Figure 4). The gene *Tango7* has previously been identified in different *Drosphila* RNAi screens as a regulator of cell viability (Boutros *et al.* 2004), as a protein involved in ERK signaling (Friedman and Perrimon 2006), Golgi function (Bard *et al.* 2006), apoptosis (Chew *et al.* 2009), lipid storage (Beller *et al.* 2008; Guo *et al.* 2008), huntingtin protein aggregation (Doumanis *et al.* 2009), and as a cell-cycle regulator (Bjorklund *et al.* 2006). Our expression profiling analysis strongly suggests that the primary function of the protein *Tango7* is as a component of the *Drosophila* eIF3/4 translation initiation complex and sequence analysis shows that *Tango7* is the ortholog of human *eIF3m*. We propose that this gene be re-named *eIF3m*.

Our results indicate that in RNAi screens that analyze a particular phenotype, genes that have a general role in cellular functions such as *Tango7* can easily be mistaken for specific regulators of the analyzed pathways or processes. In classical genetic screens, such genes are not identified as the specific phenotype is masked by the more general effect. In many cases this is desirable, as the specific phenotype is often caused by the general effect, and is therefore of little interest. We find that a combination of an RNAi screen with the analysis of a broad cellular phenotype such as an expression profile can be used to classify genes to specific and broad phenotypic classes. In addition, expression profiling allows one to control for the magnitude and specificity of the RNAi effect, thus alleviating several major weaknesses of the RNAi technology.

### Transcriptional network controlling the cell cycle

Analysis of target genes of the 23 transcription factors (based on FlyTF.org) in our screen revealed that most of the TFs had quite distinct transcriptional targets. Notable exceptions were the targets of the dimeric TFs Myc/Max and E2f/Dp, which were clearly and strongly overlapping. Loss of many TFs had a comparatively weak impact on the cell cycle, which may reflect their peripheral role in controlling cell division. The strongest effects were found for knockdown of the genes coding for *E2f/Dp*, *Myb*, *Myc/Max*, and *SREBP*.

Based on the clustering analysis, the COP9 signalosome appears to regulate the activity of the E2f transcription factors. However, only a subset of E2f targets were coregulated by COP9 signalosome components, and these genes also were regulated by loss of Cyclin A and/or Myb. Cyclin A is needed for inactivation of E2f and completion of DNA replication and S-phase (Krek *et al.* 1995), suggesting that this target gene set may be indirectly regulated by E2f. Consistently, only weak enrichment of direct E2f targets were seen in this set, and instead the genes were highly enriched in direct Myb targets (*P* < 1.1 × 10^−12^), and regulators of mitosis and cytokinesis (GO *P* < 3.8 × 10^−30^; Figure 3, top).

A GO overrepresentation analysis of the significant E2f regulated genes in our data set shows that DNA replication processes are clear E2f targets [GO *P* < 2.1 × 10^−49^; see also (van den Heuvel and Dyson 2008)]. The genes involved in DNA-replication cluster together based on their expression across all RNAi treatments (Figure 3, bottom), and as expected, are also regulated by loss of Rb and Dp. Validation of this target gene set using ChIP-seq against E2f and Dp indicated that significant fraction of the target genes are indeed directly regulated by the E2f/Dp complex (*P* < 2.7 × 10^−33^).

Taken together, our results indicate that the cell cycle has two major waves of transcription: First, the E2f/Dp-mediated regulation of genes required for DNA replication, and then a dREAM/Myb-mediated regulation of genes that are involved in mitosis and cytokinesis. The transcription factor cropped also appears to act on the latter target gene set (Figure 3, top). The two waves of transcription are in turn modulated by the Myc/Max-mediated growth response (Figure 4, top) and SREBP-mediated lipid homeostasis pathway (Figure 3, top).

We previously identified several coatomer I (COPI) components, a *Drosophila* stearoyl-CoA 9-desaturase (Desat1) and Sterol regulatory element binding protein (SREBP/HLH106) as regulators of the cell cycle (Bjorklund *et al.* 2006). In *Drosophila*, the SREBP activity is inhibited by palmitate (Seegmiller *et al.* 2002), the substrate of Desat1 (de Renobales and Blomquist 1984). Desat1, in turn, is retained in the ER by COPI proteins (Jackson *et al.* 1993; Dancourt and Barlowe 2010). Given the similarity of the expression profiles following loss of all these genes and key mitosis regulators (*e.g*., PP1-87B, microtubule star), we propose that these proteins are components of a pathway that regulates mitosis. Targets of SREBP include Myb, which controls expression of a large set of mitotic and cytokinetic regulators that includes Cdk1, polo, fzy, pav, feo, and Klp67A.

### Two cycles make two cells out of one

Perhaps the most interesting finding is that by correlating the overall effect of different RNAi treatments on cell cycle and expression levels, we found that although transcription controls the cell cycle, the progression of the cell cycle itself does not feed back to control transcription. Loss of multiple genes resulted in strong cell-cycle phenotypes without much impact on gene expression. These included *dTCF*/*Pangolin*, *MEK3*/*Licorne*, *MAPk-Ak2*, and *Myt1*, which are known or shown here to regulate the Myt1/Cdc25 Axis, decreasing tyrosine phosphorylation of Cdk1 and increasing its activity.

Treatments that caused decreased phosphorylation of Cdk1 resulted in increased G1 and decreased G2 content, without appreciable effect on transcription. This uncoupling of transcription from the cell cycle distribution of cells is surprising, particularly given the extensive literature on cell-cycle phase specific gene expression (see, for example Jensen *et al.* 2006). However, the fact that cell-cycle phase correlates with gene expression pattern does not imply that the phase itself causes the pattern. In this work, we performed an extensive set of perturbation experiments that clearly indicate that cell-cycle phase and gene expression pattern can be uncoupled from each other. We could not identify a single gene whose steady-state mRNA expression correlates highly with presence of cells in the G1 or G2 cell-cycle phases. We propose that this is due to the fact that transcription of genes is associated with transitions between cell cycle phases, not the phases themselves.

Our results suggest that the cell cycle represents two distinct but overlapping cycles, the transcriptional cycle that is responsible for production of the cellular components, and a separate assembly cycle in which the components are assembled to make two cells out of the old and new components. The mechanism we uncovered is separate from the one previously described in yeast, where it was shown that genes continue to be periodically expressed in cells where Cdk1 activity is decreased by deleting S- and M-phase−specific cyclins (Orlando *et al.* 2008). On the contrary, in *Drosophila* we found that loss of Cdk1 activity induced by RNAi treatments results in very strong transcriptional downregulation of mitotic genes (Figure 2; Figure 3), whereas gain of Cdk1 activity does not affect transcription but acts directly at protein level to effect earlier than normal entry into mitosis.

Our evidence suggests that the identified mechanism operates in the regulation of cell size; all RNAi treatments that increase G1 with limited effect on gene expression directly or indirectly regulated Cdk1 activity and resulted in decreased cell size, whereas targeting *Cdk1* itself increased cell size in G2 (Figure 6D). Increased Cdk1 activity results in smaller cells, much like what has been shown previously in yeast (Fantes and Nurse 1978; Cross 1988; Nash *et al.* 1988; Kellogg 2003). These results are consistent with a model (Figure 7B) where cell size is regulated by Cdk1 activity through the two kinases that inhibit Cdk1, the membrane bound Myt1 and the nuclear Wee1. In small cells, membrane surface and nuclear volume to cell volume ratios are high, resulting in high inhibitory kinase activity toward soluble Cdk1. When the cell grows, its cytoplasm becomes larger compared with its nucleus, and its surface to volume ratio decreases. This results in Wee1 and Myt1 becoming increasingly less effective in phosphorylating soluble Cdk1 and the amount of active Cdk1 increases, leading to mitotic entry. Possibly, levels of nuclear String are part of this regulatory system, as increased amounts of String result in smaller cells and shorter G2, whereas RNAi knockdown of *string* lead to cell cycle- and transcriptional phenotypes that are very similar to *Cdk1* knockdown. This mechanism allows for simple management of cell size in different tissues by adjusting of the amount of cellular protein of any of the units in this pathway.

## Supplementary Material

Supporting Information

## References

[bib1] Amsterdam A. Sadler K. C. Lai K. Farrington S. Bronson R. T., 2004 Many ribosomal protein genes are cancer genes in zebrafish. PLoS Biol. 2: E139 15138505 10.1371/journal.pbio.0020139 PMC406397

[bib2] Balch W. E. Morimoto R. I. Dillin A. Kelly J. W., 2008 Adapting proteostasis for disease intervention. Science 319: 916–919 18276881 10.1126/science.1141448

[bib3] Bard F. Casano L. Mallabiabarrena A. Wallace E. Saito K., 2006 Functional genomics reveals genes involved in protein secretion and Golgi organization. Nature 439: 604–607 16452979 10.1038/nature04377

[bib4] Beller M. Sztalryd C. Southall N. Bell M. Jackle H., 2008 COPI complex is a regulator of lipid homeostasis. PLoS Biol. 6: e292 19067489 10.1371/journal.pbio.0060292 PMC2586367

[bib5] Besche H. C. Peth A. Goldberg A. L., 2009 Getting to first base in proteasome assembly. Cell 138: 25–28 19596233 10.1016/j.cell.2009.06.035 PMC3824964

[bib6] Bjorklund M. Taipale M. Varjosalo M. Saharinen J. Lahdenpera J., 2006 Identification of pathways regulating cell size and cell-cycle progression by RNAi. Nature 439: 1009–1013 16496002 10.1038/nature04469

[bib7] Boutros M. Kiger A. A. Armknecht S. Kerr K. Hild M., 2004 Genome-wide RNAi analysis of growth and viability in Drosophila cells. Science 303: 832–835 14764878 10.1126/science.1091266

[bib8] Chew S. K. Chen P. Link N. Galindo K. A. Pogue K., 2009 Genome-wide silencing in Drosophila captures conserved apoptotic effectors. Nature 460: 123–127 19483676 10.1038/nature08087 PMC2777527

[bib9] Colanzi A. Corda D., 2007 Mitosis controls the Golgi and the Golgi controls mitosis. Curr. Opin. Cell Biol. 19: 386–393 17689238 10.1016/j.ceb.2007.06.002

[bib10] Conlon I. Raff M., 2003 Differences in the way a mammalian cell and yeast cells coordinate cell growth and cell-cycle progression. J. Biol. 2: 7 12733998 10.1186/1475-4924-2-7 PMC156598

[bib11] Coudreuse D. Nurse P., 2011 Driving the cell cycle with a minimal CDK control network. Nature 468: 1074–1079 21179163 10.1038/nature09543

[bib12] Cross F. R., 1988 DAF1, a mutant gene affecting size control, pheromone arrest, and cell cycle kinetics of Saccharomyces cerevisiae. Mol. Cell. Biol. 8: 4675–4684 3062366 10.1128/mcb.8.11.4675-4684.1988 PMC365557

[bib13] Dai M. Wang P. Boyd A. D. Kostov G. Athey B., 2005 Evolving gene/transcript definitions significantly alter the interpretation of GeneChip data. Nucleic Acids Res. 33: e175 16284200 10.1093/nar/gni179 PMC1283542

[bib14] Dancourt J. Barlowe C., 2010 Protein sorting receptors in the early secretory pathway. Annu. Rev. Biochem. 79: 777–802 20533886 10.1146/annurev-biochem-061608-091319

[bib15] de Renobales M. Blomquist G. J., 1984 Biosynthesis of medium chain fatty acids in Drosophila melanogaster. Arch. Biochem. Biophys. 228: 407–414 6421238 10.1016/0003-9861(84)90004-3

[bib16] Dickinson J. R., 1981 The cdc 22 mutation by *Schizosaccharomyces pombe* is a temperature-sensitive defect in nucleoside diphosphokinase. Eur. J. Biochem. 119: 341–345 6273154 10.1111/j.1432-1033.1981.tb05613.x

[bib17] Dolznig H. Grebien F. Sauer T. Beug H. Mullner E. W., 2004 Evidence for a size-sensing mechanism in animal cells. Nat. Cell Biol. 6: 899–905 15322555 10.1038/ncb1166

[bib18] Doumanis J. Wada K. Kino Y. Moore A. W. Nukina N., 2009 RNAi screening in Drosophila cells identifies new modifiers of mutant huntingtin aggregation. PLoS ONE 4: e7275 19789644 10.1371/journal.pone.0007275 PMC2748703

[bib19] Edgar B. A. O’Farrell P. H., 1989 Genetic control of cell division patterns in the Drosophila embryo. Cell 57: 177–187 2702688 10.1016/0092-8674(89)90183-9 PMC2755076

[bib20] Edgar B. A. O’Farrell P. H., 1990 The three postblastoderm cell cycles of Drosophila embryogenesis are regulated in G2 by string. Cell 62: 469–480 2199063 10.1016/0092-8674(90)90012-4 PMC2753418

[bib21] Fantes P. A. Nurse P., 1978 Control of the timing of cell division in fission yeast. Cell size mutants reveal a second control pathway. Exp. Cell Res. 115: 317–329 689088 10.1016/0014-4827(78)90286-0

[bib22] Frank J. Jr Gonzalez R. L., 2010 Structure and dynamics of a processive Brownian motor: the translating ribosome. Annu. Rev. Biochem. 79: 381–412 20235828 10.1146/annurev-biochem-060408-173330 PMC2917226

[bib23] Friedman J. H. Meulman J. J., 2004 Clustering objects on subsets of attributes. J. R. Stat. Soc., B 66: 815–849

[bib24] Friedman A. Perrimon N., 2006 A functional RNAi screen for regulators of receptor tyrosine kinase and ERK signalling. Nature 444: 230–234 17086199 10.1038/nature05280

[bib25] Gallant P. Shiio Y. Cheng P. F. Parkhurst S. M. Eisenman R. N., 1996 Myc and Max homologs in Drosophila. Science 274: 1523–1527 8929412 10.1126/science.274.5292.1523

[bib26] Giot L. Bader J. S. Brouwer C. Chaudhuri A. Kuang B., 2003 A protein interaction map of Drosophila melanogaster. Science 302: 1727–1736 14605208 10.1126/science.1090289

[bib27] Goh K. I. Cusick M. E. Valle D. Childs B. Vidal M., 2007 The human disease network. Proc. Natl. Acad. Sci. USA 104: 8685–8690 17502601 10.1073/pnas.0701361104 PMC1885563

[bib28] Goranov A. I. Cook M. Ricicova M. Ben-Ari G. Gonzalez C., 2009 The rate of cell growth is governed by cell cycle stage. Genes Dev. 23: 1408–1422 19528319 10.1101/gad.1777309 PMC2701574

[bib29] Grandori C. Mac J. Siebelt F. Ayer D. E. Eisenman R. N., 1996 Myc-Max heterodimers activate a DEAD box gene and interact with multiple E box-related sites in vivo. EMBO J. 15: 4344–4357 8861962 PMC452159

[bib30] Greasley P. J. Bonnard C. Amati B., 2000 Myc induces the nucleolin and BN51 genes: possible implications in ribosome biogenesis. Nucleic Acids Res. 28: 446–453 10606642 10.1093/nar/28.2.446 PMC102507

[bib31] Grewal S. S. Li L. Orian A. Eisenman R. N. Edgar B. A., 2005 Myc-dependent regulation of ribosomal RNA synthesis during Drosophila development. Nat. Cell Biol. 7: 295–302 15723055 10.1038/ncb1223

[bib32] Grosshans J. Wieschaus E., 2000 A genetic link between morphogenesis and cell division during formation of the ventral furrow in Drosophila. Cell 101: 523–531 10850494 10.1016/s0092-8674(00)80862-4

[bib33] Guo Y. Walther T. C. Rao M. Stuurman N. Goshima G., 2008 Functional genomic screen reveals genes involved in lipid-droplet formation and utilization. Nature 453: 657–661 18408709 10.1038/nature06928 PMC2734507

[bib34] Hachet O. Berthelot-Grosjean M. Kokkoris K. Vincenzetti V. Moosbrugger J., 2011 A phosphorylation cycle shapes gradients of the DYRK family kinase Pom1 at the plasma membrane. Cell 145: 1116–1128 21703453 10.1016/j.cell.2011.05.014

[bib35] Hartwell L. H. Mortimer R. K. Culotti J. Culotti M., 1973 Genetic control of the cell division cycle in yeast: V. Genetic analysis of *cdc* mutants. Genetics 74: 267–286 17248617 10.1093/genetics/74.2.267 PMC1212945

[bib36] Holstege F. C. Jennings E. G. Wyrick J. J. Lee T. I. Hengartner C. J., 1998 Dissecting the regulatory circuitry of a eukaryotic genome. Cell 95: 717–728 9845373 10.1016/s0092-8674(00)81641-4

[bib37] Hughes T. R. Marton M. J. Jones A. R. Roberts C. J. Stoughton R., 2000 Functional discovery via a compendium of expression profiles. Cell 102: 109–126 10929718 10.1016/s0092-8674(00)00015-5

[bib38] Irizarry R. A. Hobbs B. Collin F. Beazer-Barclay Y. D. Antonellis K. J., 2003 Exploration, normalization, and summaries of high density oligonucleotide array probe level data. Biostatistics 4: 249–264 12925520 10.1093/biostatistics/4.2.249

[bib39] Jackson M. R. Nilsson T. Peterson P. A., 1993 Retrieval of transmembrane proteins to the endoplasmic reticulum. J. Cell Biol. 121: 317–333 8468349 10.1083/jcb.121.2.317 PMC2200111

[bib40] Jensen L. J. Jensen T. S. de Lichtenberg U. Brunak S. Bork P., 2006 Co-evolution of transcriptional and post-translational cell-cycle regulation. Nature 443: 594–597 17006448 10.1038/nature05186

[bib41] Jin Z. Homola E. Tiong S. Campbell S. D., 2008 Drosophila myt1 is the major cdk1 inhibitory kinase for wing imaginal disc development. Genetics 180: 2123–2133 18940789 10.1534/genetics.108.093195 PMC2600946

[bib42] Jorgensen P. Rupes I. Sharom J. R. Schneper L. Broach J. R., 2004 A dynamic transcriptional network communicates growth potential to ribosome synthesis and critical cell size. Genes Dev. 18: 2491–2505 15466158 10.1101/gad.1228804 PMC529537

[bib43] Kastan M. B. Bartek J., 2004 Cell-cycle checkpoints and cancer. Nature 432: 316–323 15549093 10.1038/nature03097

[bib44] Kellogg D. R., 2003 Wee1-dependent mechanisms required for coordination of cell growth and cell division. J. Cell Sci. 116: 4883–4890 14625382 10.1242/jcs.00908

[bib45] Kivioja T. Vähärautio A. Karlsson K. Bonke M. Linnarsson S., 2011 Counting absolute number of molecules using unique molecular identifiers. Nat. Preced. 20: 72–74 10.1038/nmeth.1778 22101854

[bib46] Koepp D. M. Harper J. W. Elledge S. J., 1999 How the cyclin became a cyclin: regulated proteolysis in the cell cycle. Cell 97: 431–434 10338207 10.1016/s0092-8674(00)80753-9

[bib47] Kongsuwan K. Yu Q. Vincent A. Frisardi M. C. Rosbash M., 1985 A Drosophila Minute gene encodes a ribosomal protein. Nature 317: 555–558 4047173 10.1038/317555a0

[bib48] Korenjak M. Taylor-Harding B. Binne U. K. Satterlee J. S. Stevaux O., 2004 Native E2F/RBF complexes contain Myb-interacting proteins and repress transcription of developmentally controlled E2F target genes. Cell 119: 181–193 15479636 10.1016/j.cell.2004.09.034

[bib49] Krek W. Xu G. Livingston D. M., 1995 Cyclin A-kinase regulation of E2F–1 DNA binding function underlies suppression of an S phase checkpoint. Cell 83: 1149–1158 8548802 10.1016/0092-8674(95)90141-8

[bib50] Lee M. G. Norbury C. J. Spurr N. K. Nurse P., 1988 Regulated expression and phosphorylation of a possible mammalian cell-cycle control protein. Nature 333: 676–679 3287181 10.1038/333676a0

[bib51] Lehman D. A. Patterson B. Johnston L. A. Balzer T. Britton J. S., 1999 Cis-regulatory elements of the mitotic regulator, string/Cdc25. Development 126: 1793–1803 10101114 10.1242/dev.126.9.1793 PMC10176497

[bib52] Lehner C. F. O’Farrell P. H., 1990 Drosophila cdc2 homologs: a functional homolog is coexpressed with a cognate variant. EMBO J. 9: 3573–3581 2120045 10.1002/j.1460-2075.1990.tb07568.x PMC552108

[bib53] Lewis P. W. Beall E. L. Fleischer T. C. Georlette D. Link A. J., 2004 Identification of a Drosophila Myb-E2F2/RBF transcriptional repressor complex. Genes Dev. 18: 2929–2940 15545624 10.1101/gad.1255204 PMC534653

[bib54] Li M. Z. Elledge S. J., 2005 MAGIC, an in vivo genetic method for the rapid construction of recombinant DNA molecules. Nat. Genet. 37: 311–319 15731760 10.1038/ng1505

[bib55] Lipsick J. S., 2004 synMuv verite–Myb comes into focus. Genes Dev. 18: 2837–2844 15574590 10.1101/gad.1274804

[bib56] Lundgren J. Masson P. Mirzaei Z. Young P., 2005 Identification and characterization of a Drosophila proteasome regulatory network. Mol. Cell. Biol. 25: 4662–4675 15899868 10.1128/MCB.25.11.4662-4675.2005 PMC1140619

[bib57] Manke I. A. Nguyen A. Lim D. Stewart M. Q. Elia A. E., 2005 MAPKAP kinase-2 is a cell cycle checkpoint kinase that regulates the G2/M transition and S phase progression in response to UV irradiation. Mol. Cell 17: 37–48 15629715 10.1016/j.molcel.2004.11.021

[bib58] Martin S. G. Berthelot-Grosjean M., 2009 Polar gradients of the DYRK-family kinase Pom1 couple cell length with the cell cycle. Nature 459: 852–856 19474792 10.1038/nature08054

[bib59] Marygold S. J. Roote J. Reuter G. Lambertsson A. Ashburner M., 2007 The ribosomal protein genes and Minute loci of *Drosophila melanogaster*. Genome Biol. 8: R216 17927810 10.1186/gb-2007-8-10-r216 PMC2246290

[bib60] Moseley J. B. Mayeux A. Paoletti A. Nurse P., 2009 A spatial gradient coordinates cell size and mitotic entry in fission yeast. Nature 459: 857–860 19474789 10.1038/nature08074

[bib61] Mueller P. R. Coleman T. R. Kumagai A. Dunphy W. G., 1995 Myt1: a membrane-associated inhibitory kinase that phosphorylates Cdc2 on both threonine-14 and tyrosine-15. Science 270: 86–90 7569953 10.1126/science.270.5233.86

[bib62] Murray A. W., 2004 Recycling the cell cycle: cyclins revisited. Cell 116: 221–234 14744433 10.1016/s0092-8674(03)01080-8

[bib63] Musacchio A. Salmon E. D., 2007 The spindle-assembly checkpoint in space and time. Nat. Rev. Mol. Cell Biol. 8: 379–393 17426725 10.1038/nrm2163

[bib64] Narla A. Ebert B. L., 2010 Ribosomopathies: human disorders of ribosome dysfunction. Blood 115: 3196–3205 20194897 10.1182/blood-2009-10-178129 PMC2858486

[bib65] Nash R. Tokiwa G. Anand S. Erickson K. Futcher A. B., 1988 The WHI1+ gene of Saccharomyces cerevisiae tethers cell division to cell size and is a cyclin homolog. EMBO J. 7: 4335–4346 2907481 10.1002/j.1460-2075.1988.tb03332.x PMC455150

[bib66] Nurse P. Thuriaux P. Nasmyth K., 1976 Genetic control of the cell division cycle in the fission yeast *Schizosaccharomyces pombe*. Mol. Gen. Genet. 146: 167–178 958201 10.1007/BF00268085

[bib67] Orlando D. A. Lin C. Y. Bernard A. Wang J. Y. Socolar J. E., 2008 Global control of cell-cycle transcription by coupled CDK and network oscillators. Nature 453: 944–947 18463633 10.1038/nature06955 PMC2736871

[bib68] Park K. Millet L. J. Kim N. Li H. Jin X., 2010 Measurement of adherent cell mass and growth. Proc. Natl. Acad. Sci. USA 107: 20691–20696 21068372 10.1073/pnas.1011365107 PMC2996435

[bib69] Pesin J. A. Orr-Weaver T. L., 2008 Regulation of APC/C activators in mitosis and meiosis. Annu. Rev. Cell Dev. Biol. 24: 475–499 18598214 10.1146/annurev.cellbio.041408.115949 PMC4070676

[bib70] Pilkinton M. Sandoval R. Colamonici O. R., 2007 Mammalian Mip/LIN-9 interacts with either the p107, p130/E2F4 repressor complex or B-Myb in a cell cycle-phase-dependent context distinct from the Drosophila dREAM complex. Oncogene 26: 7535–7543 17563750 10.1038/sj.onc.1210562

[bib71] Rajalingam K. Dikic I., 2011 Healthy ageing through regulated proteostasis. EMBO J. 30: 2983–2985 21811299 10.1038/emboj.2011.237 PMC3160197

[bib72] Reddy B. A. Bajpe P. K. Bassett A. Moshkin Y. M. Kozhevnikova E., 2010 Drosophila transcription factor Tramtrack69 binds MEP1 to recruit the chromatin remodeler NuRD. Mol. Cell. Biol. 30: 5234–5244 20733004 10.1128/MCB.00266-10 PMC2953047

[bib73] Reed S. I., 1980 The selection of S. cerevisiae mutants defective in the start event of cell division. Genetics 95: 561–577 7002718 10.1093/genetics/95.3.561 PMC1214247

[bib74] Rustici G. Mata J. Kivinen K. Lio P. Penkett C. J., 2004 Periodic gene expression program of the fission yeast cell cycle. Nat. Genet. 36: 809–817 15195092 10.1038/ng1377

[bib75] Santamaria D. Barriere C. Cerqueira A. Hunt S. Tardy C., 2007 Cdk1 is sufficient to drive the mammalian cell cycle. Nature 448: 811–815 17700700 10.1038/nature06046

[bib76] Schneider I., 1972 Cell lines derived from late embryonic stages of Drosophila melanogaster. J. Embryol. Exp. Morphol. 27: 353–365 4625067

[bib77] Seegmiller A. C. Dobrosotskaya I. Goldstein J. L. Ho Y. K. Brown M. S., 2002 The SREBP pathway in Drosophila: regulation by palmitate, not sterols. Dev. Cell 2: 229–238 11832248 10.1016/s1534-5807(01)00119-8

[bib78] Shannon P. Markiel A. Ozier O. Baliga N. S. Wang J. T., 2003 Cytoscape: a software environment for integrated models of biomolecular interaction networks. Genome Res. 13: 2498–2504 14597658 10.1101/gr.1239303 PMC403769

[bib79] Sherlock G., 2000 Analysis of large-scale gene expression data. Curr. Opin. Immunol. 12: 201–205 10712947 10.1016/s0952-7915(99)00074-6

[bib80] Smyth G. K., 2004 Linear models and empirical bayes methods for assessing differential expression in microarray experiments. Stat. Appl. Genet. Mol. Biol. 3: Article3 16646809 10.2202/1544-6115.1027

[bib81] Son S. Tzur A. Weng Y. Jorgensen P. Kim J., 2012 Direct observation of mammalian cell growth and size regulation. Nat. Methods 9: 910–912 22863882 10.1038/nmeth.2133 PMC3433595

[bib82] Tuupanen S. Turunen M. Lehtonen R. Hallikas O. Vanharanta S., 2009 The common colorectal cancer predisposition SNP rs6983267 at chromosome 8q24 confers potential to enhanced Wnt signaling. Nat. Genet. 41: 885–890 19561604 10.1038/ng.406

[bib83] Tzur A. Kafri R. LeBleu V. S. Lahav G. Kirschner M. W., 2009 Cell growth and size homeostasis in proliferating animal cells. Science 325: 167–171 19589995 10.1126/science.1174294 PMC2905160

[bib84] van den Heuvel S. Dyson N. J., 2008 Conserved functions of the pRB and E2F families. Nat. Rev. Mol. Cell Biol. 9: 713–724 18719710 10.1038/nrm2469

[bib85] van Riggelen J. Yetil A. Felsher D. W., 2010 MYC as a regulator of ribosome biogenesis and protein synthesis. Nat. Rev. Cancer 10: 301–309 20332779 10.1038/nrc2819

[bib86] Wei N. Serino G. Deng X. W., 2008 The COP9 signalosome: more than a protease. Trends Biochem. Sci. 33: 592–600 18926707 10.1016/j.tibs.2008.09.004

[bib87] Weigmann K. Cohen S. M. Lehner C. F., 1997 Cell cycle progression, growth and patterning in imaginal discs despite inhibition of cell division after inactivation of Drosophila Cdc2 kinase. Development 124: 3555–3563 9342048 10.1242/dev.124.18.3555

[bib88] Whitfield M. L. Sherlock G. Saldanha A. J. Murray J. I. Ball C. A., 2002 Identification of genes periodically expressed in the human cell cycle and their expression in tumors. Mol. Biol. Cell 13: 1977–2000 12058064 10.1091/mbc.02-02-0030. PMC117619

[bib89] Xie Y. Varshavsky A., 2001 RPN4 is a ligand, substrate, and transcriptional regulator of the 26S proteasome: a negative feedback circuit. Proc. Natl. Acad. Sci. USA 98: 3056–3061 11248031 10.1073/pnas.071022298 PMC30606

[bib90] Zaffran S. Chartier A. Gallant P. Astier M. Arquier N., 1998 A Drosophila RNA helicase gene, pitchoune, is required for cell growth and proliferation and is a potential target of d-Myc. Development 125: 3571–3584 9716523 10.1242/dev.125.18.3571

