# Supplementary material for: Transcriptional Networks Controlling the Cell Cycle
Source: G3 (Bethesda). 2013 Jan 1;3(1):75–90. doi: 10.1534/g3.112.004283 (PMC3538345; doi:10.1534/g3.112.004283)
Supplement: Supporting Information [file supp_3_1_75__index.html]

Supporting Information 

# Transcriptional Networks Controlling the Cell Cycle

## Supporting Information for Bonke *et al.*, 2013

**Files in this Data Supplement:**

- Supporting Information - Figures S1-S7, Files S1-S7, and Tables S1-S12 (PDF, 2 MB)
- Figure S1 - Two-dimensional clustering of the RNA-seq derived expression profiling data (PDF, 178 KB)
- Figure S2 - Targeted genes are downregulated by RNAi (PDF, 113 KB)
- Figure S3 - A Normalization of the array data. B Overlap percentage plot of the significant genes in all experiments. (PDF, 305 KB)
- Figure S4 - Overlap between significantly regulated target genes in all samples (PDF, 307 KB)
- Figure S5 - A Network representation of the overlap between target genes that were significantly regulated in the different RNAi treatments. B LTQ mass-spec. (PDF, 493 KB)
- Figure S6 - Transcriptional network regulating the cell cycle (PDF, 917 KB)
- Figure S7 - A Overlap between target genes of *Myt1, MAPk-Ak2, Pan/dTCF* and *Lic/MEK3*. B Relative amount of unphosphorylated Cdk1, Cdk1-Cdk1P in *Drosophila* S2 cells as a function of cell size, FSC-A. (PDF, 99 KB)
- File S1
- File S1 - Cytoscape network representation of Figure S5A (.cys, 67 KB)
- File S2 - Cytoscape network representation of Figure S6 (.cys, 90 KB)
- File S3 - Cytoscape network representation of Figure 3 (top) (.cys, 63 KB)
- File S4 - Cytoscape network representation of Figure 3 (bottom) (.cys, 40 KB)
- File S5 - Cytoscape network representation of Figure 4 (top) (.cys, 65 KB)
- File S6 - Cytoscape network representation of Figure 4 (bottom) (.cys, 48 KB)
- File S7 - Data file containing the entire dataset, (Limma R Bioconductor output format) (.zip, 74.6 MB)
- Table S1 - Flow cytometry phenotypes (.xlsx, 18 KB)
- Table S2 - Significantly regulated genes for each experiment (.xlsx, 4.2 MB)
- Table S3 - qPCR validation of RNAi (.xlsx, 10 KB)
- Table S4 - Comparison of data between our results and Reddy et al., 2010 (.xlsx, 14 KB)
- Table S5 - Gene overlap analysis of experiments (.xlsx, 56 KB)
- Table S6 - Significantly overrepresented GO annotations for each experiment (.xlsx, 480 KB)
- Table S7 - Significantly overrepresented GO annotations for each of the clusters of Figure 4 (.xlsx, 72 KB)
- Table S8 - Correlation between phenotype and expression (.xlsx, 18 KB)
- Table S9 - Correlation between phenotype and expression, associated GO overrepresentation of correlating genes (.xlsx, 27 KB)
- Table S10 - dsRNA constructs (.xlsx, 14 KB)
- Table S11 - Fly to Human orthologues (.xlsx, 9 KB)
- Table S12 - GO annotations (.xlsx, 61 KB)
