# Supplementary material for: Transcriptional Networks Controlling the Cell Cycle
Source: G3 (Bethesda). 2013 Jan 1;3(1):75–90. doi: 10.1534/g3.112.004283 (PMC3538345; doi:10.1534/g3.112.004283)
Supplement: Supporting Information [file supp_3.1.75_004283SI.pdf]

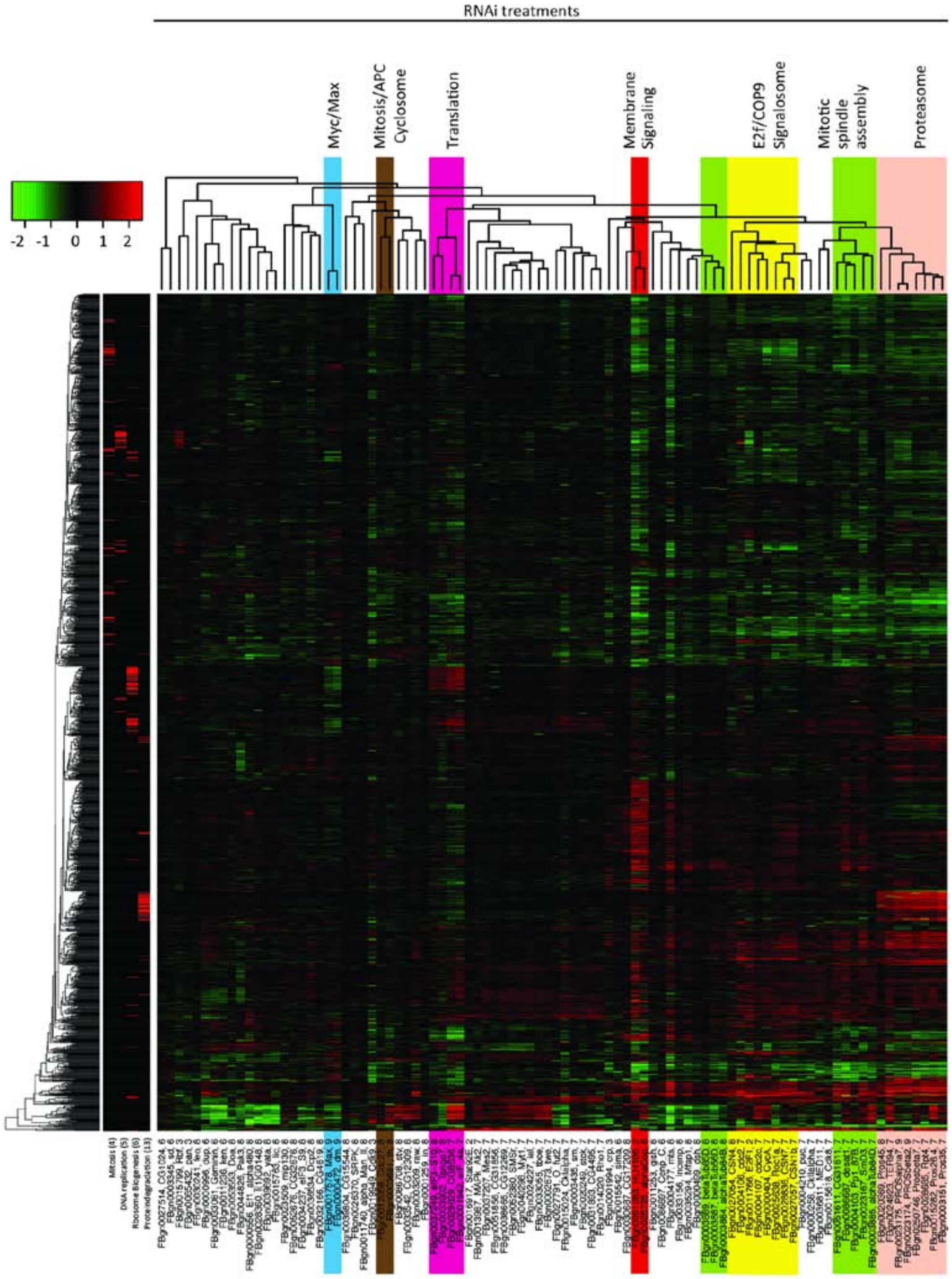

Figure S1 Two-dimensional clustering of the RNA-seq derived expression profiling data. Samples and target genes were clustered using Pearson and COSA, lambda = 0.6, average linkage distance metrics, respectively, based on expression of 1470 genes that were significantly regulated in one or more samples, > 1 absolute log2 fold change, p < 0.01). Sample subclusters are named as in **Figure 2**, and indicated by coloring the corresponding branch of the dendrogram. Note that the sample subclusters are similar to those obtained using array-based expression profiling, compare to **Figure 2**). The gene clustering analysis also resulted in similar clusters; positions of the genes from the the mitosis, DNA replication, ribosome biogenesis and protein degradation clusters from array-based clustering are shown on the left heatmap, red bars). Sample name and RNAi and RNA extraction batch number in which the sample was processed are indicated on the bottom.

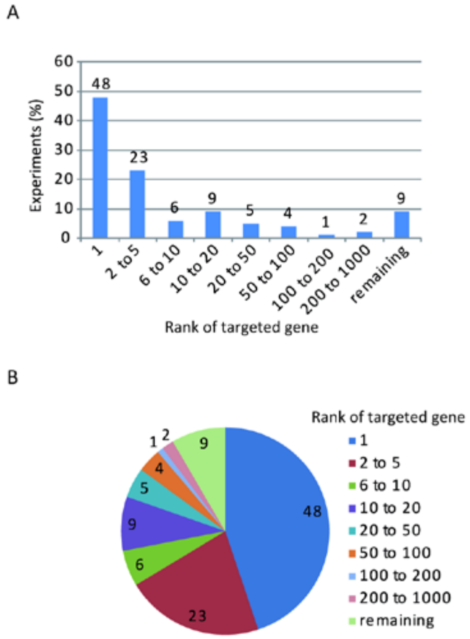

Figure S2 Targeted genes are downregulated by RNAi. (A) Column chart representing the rank of targeted genes within the experiments. The numbers above each bar represent the number of experiments that were represented within the interval of that bar. Note that in 48 of the 111 experiments, including all repeats of *Myb* and *Trx* the targeted gene was the strongest downregulated gene. (B) Pie chart representing the same data as in, (A).

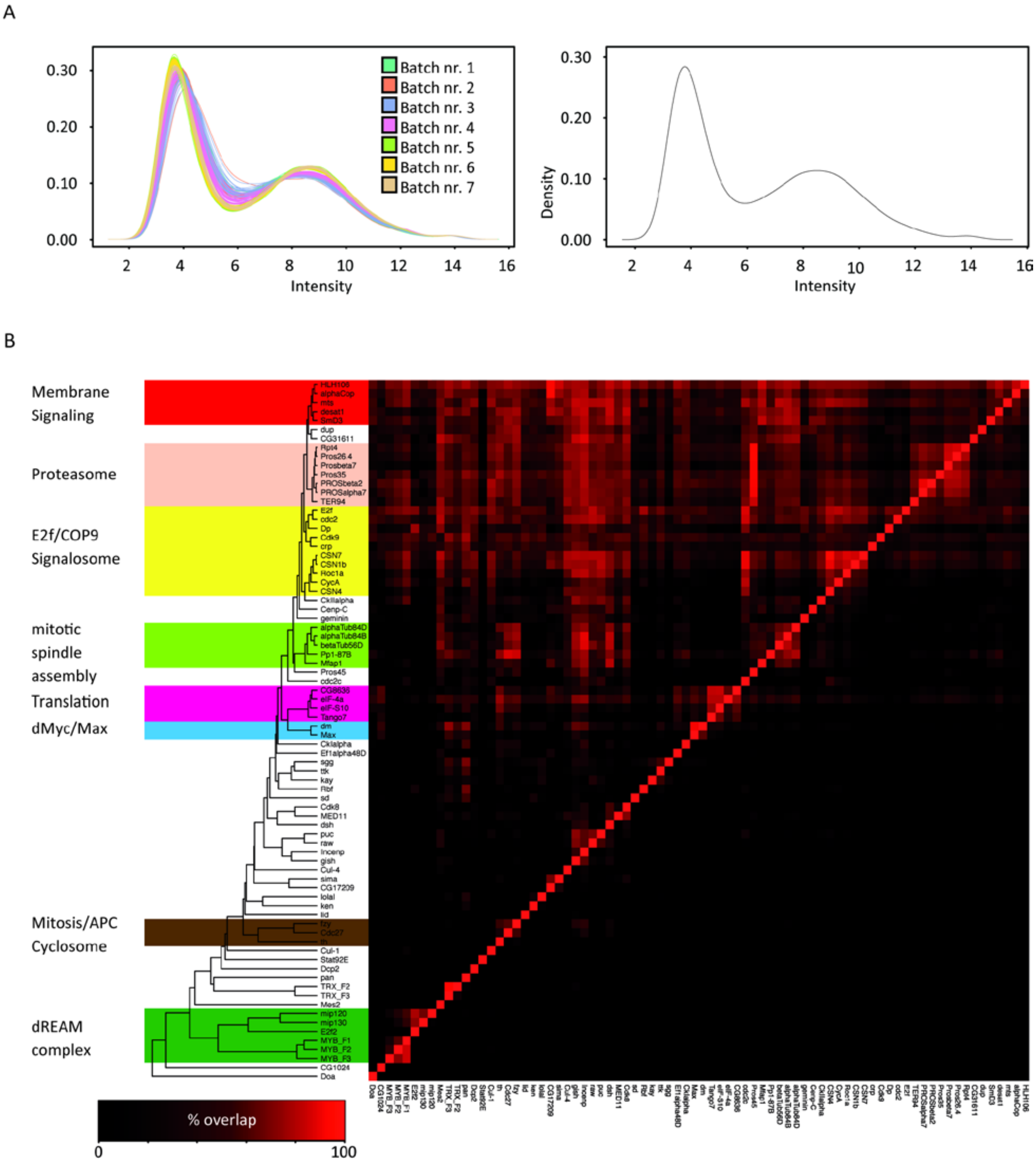

Figure S3 (A) Normalization of the array data. Left: Histogram of the log_2 expression values from all *Drosophila* genome 2.0 arrays after RMA normalization. Samples are colored according to the batch in which they were processed. Note that the distribution of the expression values is batch-dependent. Right: Histograms after correction of the batch effect using quantile normalization. (B) Overlap percentage plot of the significant genes in all experiments. For each experiment with more than 50 significantly regulated genes, absolute logfold change > 0.3 at a p-value < 0.01), the percentage of overlap was calculated with the significantly regulated genes of all other experiments matching these criteria. Clustering was performed using the cosine angle distance metric and hierarchical clustering used average linkage. Diagonal shows self-overlap, 100%).

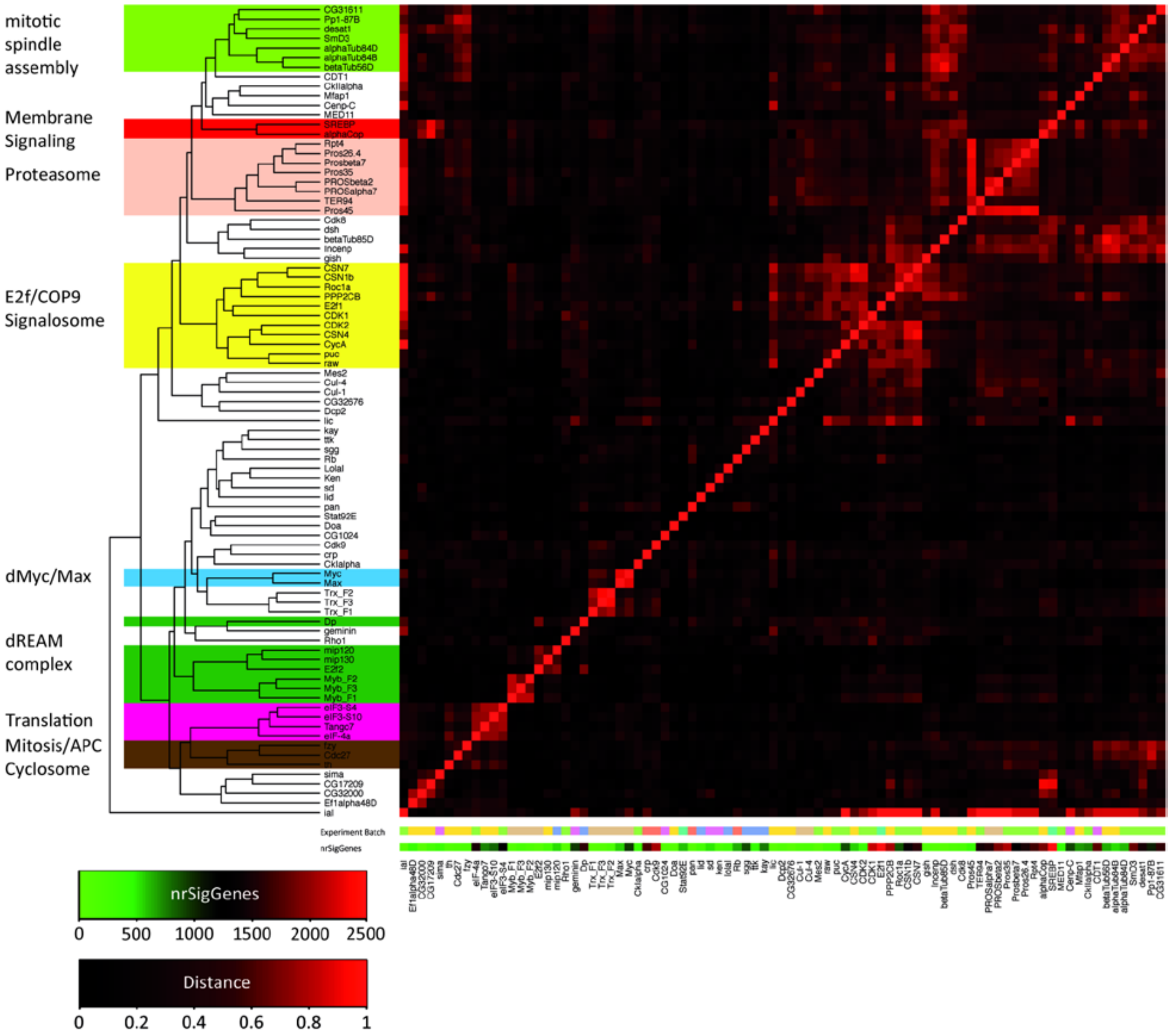

Figure S4 Overlap between significantly regulated target genes in all samples. Excess gene overlap over that expected by random was calculated for each pair of expression analyses in the same and in the opposite directions. The overlap scores for up- and down-regulated genes was calculated and the higher of the two was used in hierarchical clusterering and plotted as a heatmap. Subclusters that contain multiple members of the same protein complex or signaling pathway are named according to the known function of the genes, and indicated by coloring the corresponding branch of the dendrogram similar to the colors used in **Figure 2**.

A

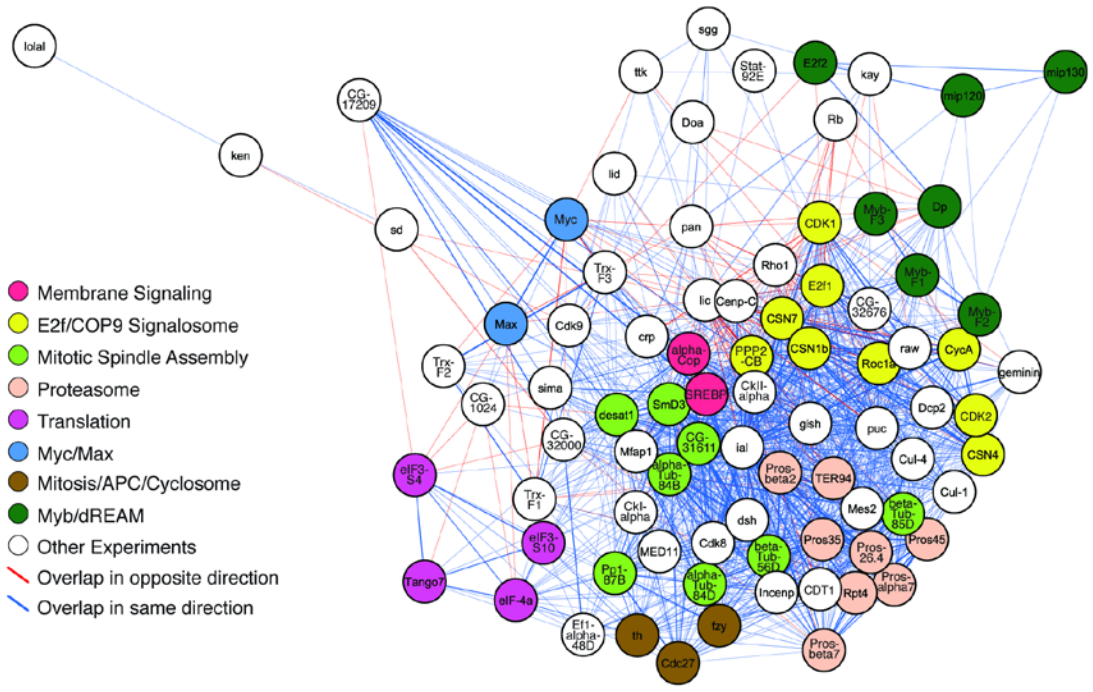

B

					PEPTIDES IDENTIFIED	
Rank	Gene ID	FBgn	Enrichment score	Peptides in S2 lysate	Replicate 1	Replicate 2
1	lolal	FBgn0022238	8.85	13	47	68
2	RpS14a	FBgn0004403	2.00	ND	0	1
3	RpS19a	FBgn0010412	2.00	ND	0	1
4	alphaTub84B	FBgn0003884	1.33	12	7	9
5	ana3	FBgn0033718	1.00	1	0	1
6	ken	FBgn0011236	1.00	4	0	4
7	CG9791	FBgn0037232	1.00	2	2	0
8	CG2064	FBgn0033205	0.58	19	6	5
9	CG11377	FBgn0031217	0.57	7	0	4
10	ttk	FBgn0003870	0.33	6	0	2

Figure S5 (A) Network representation of the overlap between target genes that were significantly regulated in the different RNAi treatments. Overlap exceeding that expected by random selection of genes was determined for each RNAi treatment pair. Gene sets regulated in the same and opposite directions were considered separately, and the direction displaying higher overlap was selected. Nodes representing samples are connected to each other by an edge if they have excess overlap, see Methods for details). Thickness of the edge represents the level of overlap, and color indicates whether the overlapping genes were regulated in the same, blue or opposite, red direction. Network is laid out using Cytoscape, Shannon et al., 2003 yFiles organic layout algorithm. Nodes are colored according to the color scheme from **Figure 2**. Note that a different bioinformatic method used here results in a very similar classification of the samples. Overlap analysis also classifies samples that have very few regulated genes, e.g. *E2f2*; dark green and identifies connections between proteins that are found in different complexes, such as the dREAM complex, dark green that consists of *mip120*, *mip130* and either *Myb* or *E2f2*/*DP*. A heatmap of the same data classified by hierarchical clustering is shown in **Figure S4**. (B) LTQ mass-spec. Lolal protein was purified using tandem affinity purification from stable S2 cell line expressing relatively low levels of lolal protein tagged with streptavidin binding protein and hexahistidine tags, Turunen et al. 2012, in preparation). Proteins are ranked by enrichment ratio, number of peptides in sample divided by number of peptides in S2 cell lysate;, Brunner et al., 2007)). The Ken protein was among the ten most enriched proteins shown.

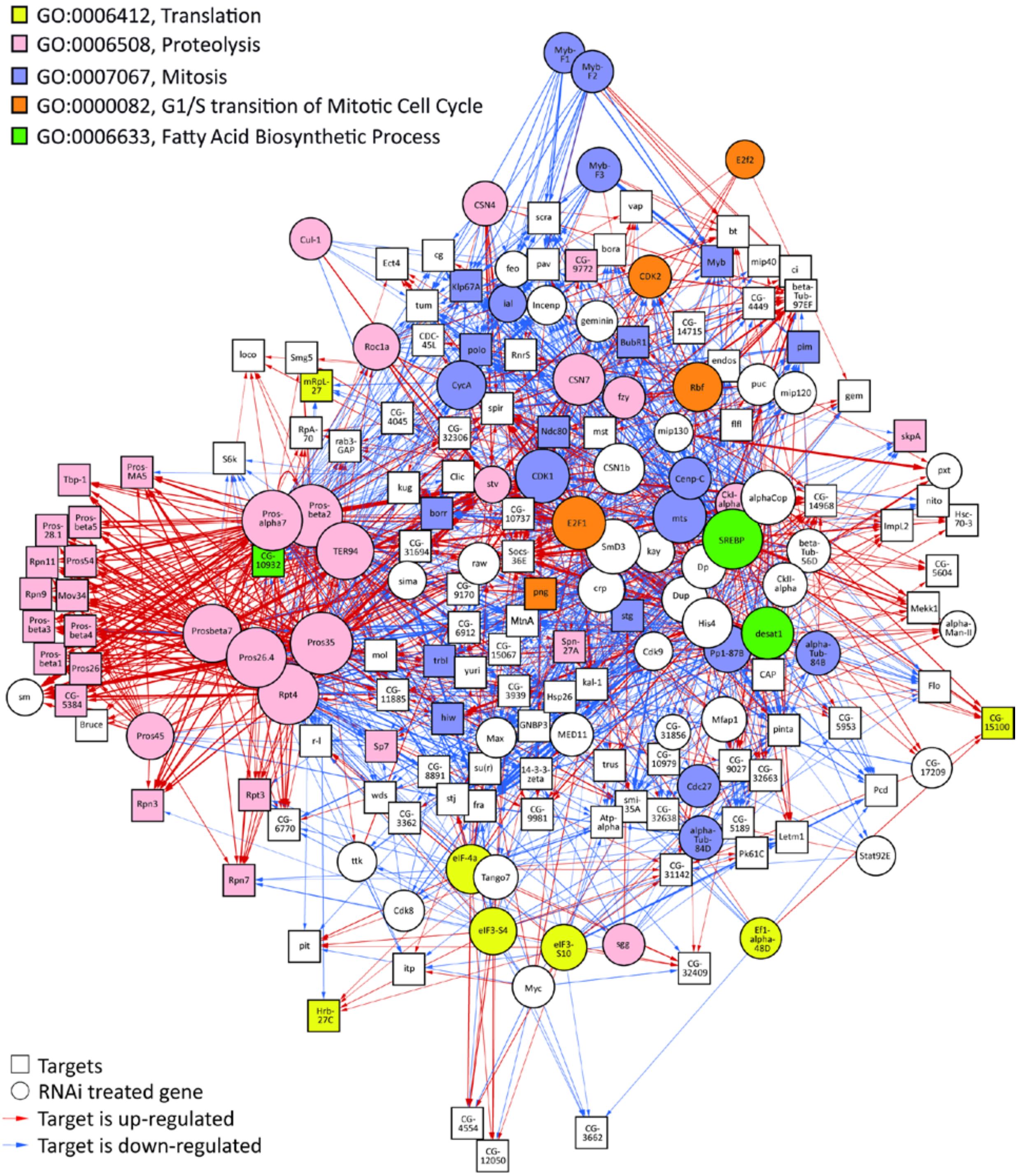

Figure S6 Transcriptional network regulating the cell cycle. Nodes are connected by an edge if a RNAi treated gene, circle results in a significant regulation of a target gene, box). Thickness of the edge represents the magnitude of the effect, and its color indicates upregulation, red or downregulation, blue of a target gene after RNAi. Size of the circles indicates the number of target genes regulated. Nodes are colored according to GO annotations indicated in the inset. Only target genes whose loss affects cell cycle or cell size in S2 cells, Bjorklund et al., 2006); Björklund et al., in preparation are included in the network. Network is laid out using yFiles organic algorithm.

A

	Myt1	Mapk-Ak2	Pan (dTCF)	Lic (dMEK2)
Myt1	1			
Mapk-Ak2	0	2		
Pan (dTCF)	0	0	64	
Lic (dMEK2)	0	1	0	6

B

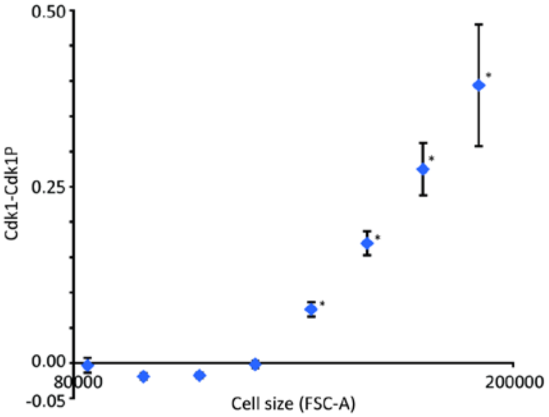

Figure S7 (A) Overlap between target genes of *Myt1*, *MAPk-Ak2*, *Pan/dTCF* and *Lic/MEK3*. Numbers indicate shared target genes. Note that no target gene is common to all of these samples. (B) Relative amount of unphosphorylated Cdk1, Cdk1-Cdk1P in *Drosophila S2* cells as a function of cell size, FSC-A). Note that unphosphorylated Cdk1 rapidly increases after the G2 cells reach a certain size. Error bars indicate one standard error, and asterisks p < 0.01, Kolmogorov-Smirnov test).

Files S1-S7

Available for download at http://www.g3journal.org/lookup/suppl/doi:10.1534/g3.112.004283/-/DC1

File S1: Cytoscape network representation of Figure S5A

File S2: Cytoscape network representation of Figure S6

File S3: Cytoscape network representation of Figure 3 (top)

File S4: Cytoscape network representation of Figure 3 (bottom)

File S5: Cytoscape network representation of Figure 4 (top)

File S6: Cytoscape network representation of Figure 4 (bottom)

File S7: Data file containing the entire dataset (Limma R Bioconductor output format)

Tables S1-S12

Available for download at http://www.g3journal.org/lookup/suppl/doi:10.1534/g3.112.004283/-/DC1

Table S1: Flow cytometry phenotypes

Table S2: Significantly regulated genes for each experiment

Table S3: qPCR validation of RNAi

Table S4: Comparison of data between our results and Reddy *et al.* (2010)

Table S5: Gene overlap analysis of experiments

Table S6: Significantly overrepresented GO annotations for each experiment

Table S7: Significantly overrepresented GO annotations for each of the clusters of Figure 2

Table S8: Correlation between phenotype and expression

Table S9: Correlation between phenotype and expression, associated GO overrepresentation of correlating genes

Table S10: dsRNA constructs

Table S11: Fly to Human orthologues

Table S12: GO annotations
