# Supplementary material for: Transcriptional Networks Controlling the Cell Cycle
Source: G3 (Bethesda). 2013 Jan 1;3(1):75–90. doi: 10.1534/g3.112.004283 (PMC3538345; doi:10.1534/g3.112.004283)
Supplement: Supporting Information [file supp_3.1.75_FigureS1.pdf]

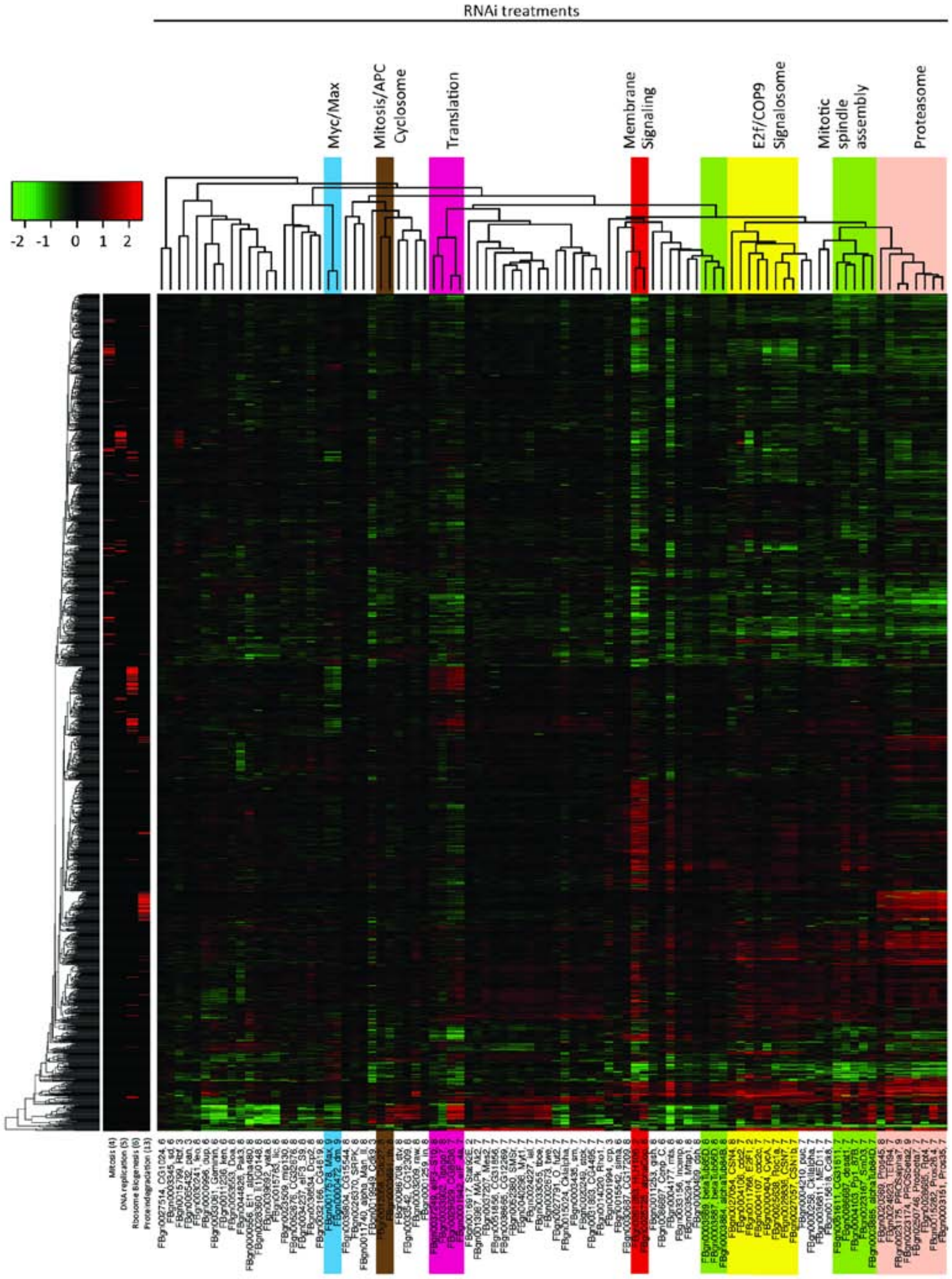

Figure S1 Two-dimensional clustering of the RNA-seq derived expression profiling data. Samples and target genes were clustered using Pearson and COSA, lambda = 0.6, average linkage distance metrics, respectively, based on expression of 1470 genes that were significantly regulated in one or more samples, > 1 absolute log2 fold change, $p < 0.01$). Sample subclusters are named as in **Figure 2**, and indicated by coloring the corresponding branch of the dendrogram. Note that the sample subclusters are similar to those obtained using array-based expression profiling, compare to **Figure 2**). The gene clustering analysis also resulted in similar clusters; positions of the genes from the the mitosis, DNA replication, ribosome biogenesis and protein degradation clusters from array-based clustering are shown on the left heatmap, red bars). Sample name and RNAi and RNA extraction batch number in which the sample was processed are indicated on the bottom.
