# Supplementary material for: Transcriptional Networks Controlling the Cell Cycle
Source: G3 (Bethesda). 2013 Jan 1;3(1):75–90. doi: 10.1534/g3.112.004283 (PMC3538345; doi:10.1534/g3.112.004283)
Supplement: Supporting Information [file supp_3.1.75_FigureS2.pdf]

A

B

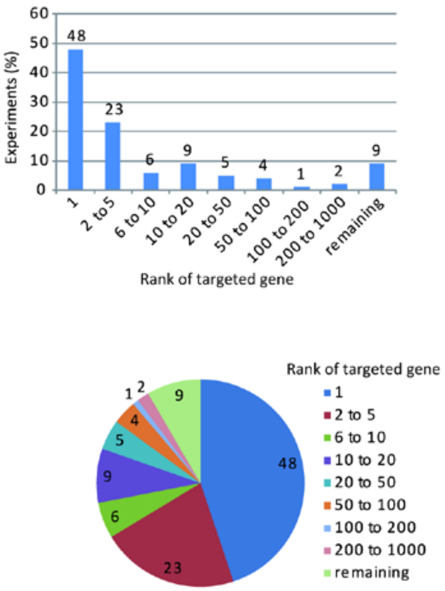

Figure S2 Targeted genes are downregulated by RNAi. (A) Column chart representing the rank of targeted genes within the experiments. The numbers above each bar represent the number of experiments that were represented within the interval of that bar. Note that in 48 of the 111 experiments, including all repeats of *Myb* and *Trx* the targeted gene was the strongest downregulated gene. (B) Pie chart representing the same data as in, (A).
