# Supplementary material for: Transcriptional Networks Controlling the Cell Cycle
Source: G3 (Bethesda). 2013 Jan 1;3(1):75–90. doi: 10.1534/g3.112.004283 (PMC3538345; doi:10.1534/g3.112.004283)
Supplement: Supporting Information [file supp_3.1.75_FigureS3.pdf]

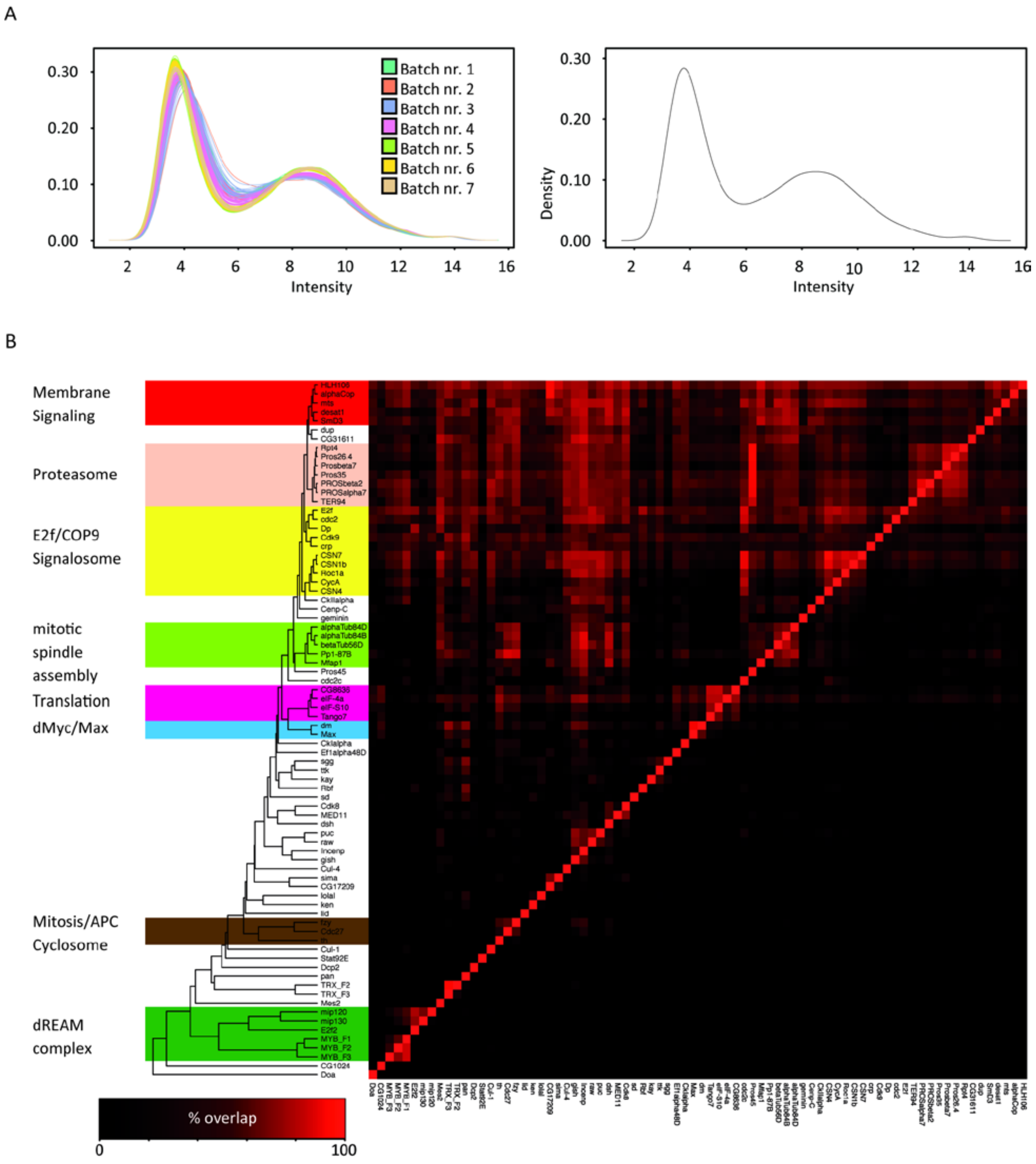

Figure S3 (A) Normalization of the array data. Left: Histogram of the log_2 expression values from all *Drosophila* genome 2.0 arrays after RMA normalization. Samples are colored according to the batch in which they were processed. Note that the distribution of the expression values is batch-dependent. Right: Histograms after correction of the batch effect using quantile normalization. (B) Overlap percentage plot of the significant genes in all experiments. For each experiment with more than 50 significantly regulated genes, absolute logfold change > 0.3 at a p-value < 0.01), the percentage of overlap was calculated with the significantly regulated genes of all other experiments matching these criteria. Clustering was performed using the cosine angle distance metric and hierarchical clustering used average linkage. Diagonal shows self-overlap, 100%).
