# Supplementary material for: Transcriptional Networks Controlling the Cell Cycle
Source: G3 (Bethesda). 2013 Jan 1;3(1):75–90. doi: 10.1534/g3.112.004283 (PMC3538345; doi:10.1534/g3.112.004283)
Supplement: Supporting Information [file supp_3.1.75_FigureS4.pdf]

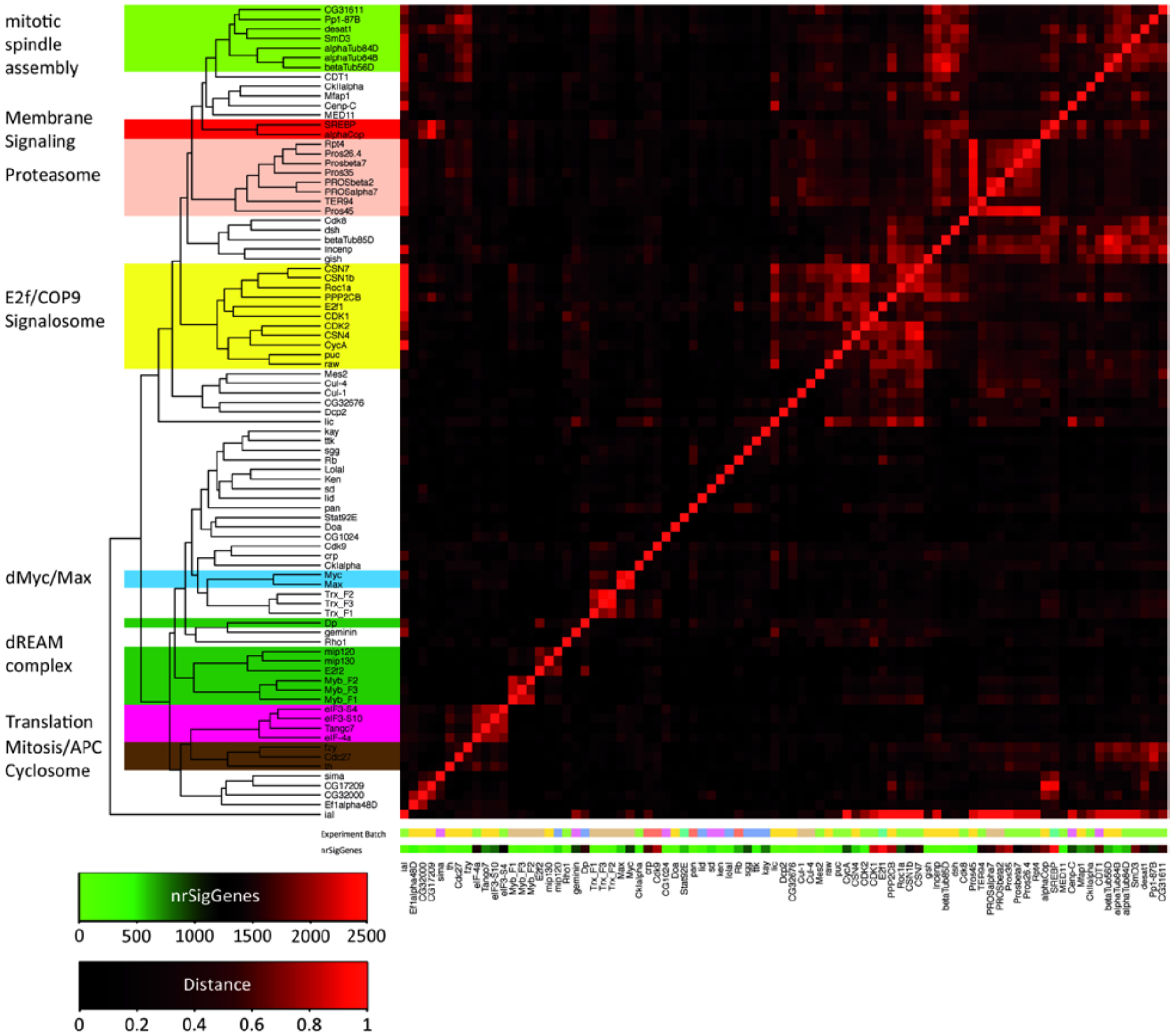

Figure S4 Overlap between significantly regulated target genes in all samples. Excess gene overlap over that expected by random was calculated for each pair of expression analyses in the same and in the opposite directions. The overlap scores for up- and down-regulated genes was calculated and the higher of the two was used in hierarchical clusterering and plotted as a heatmap. Subclusters that contain multiple members of the same protein complex or signaling pathway are named according to the known function of the genes, and indicated by coloring the corresponding branch of the dendrogram similar to the colors used in **Figure 2**.
