# Supplementary material for: Transcriptional Networks Controlling the Cell Cycle
Source: G3 (Bethesda). 2013 Jan 1;3(1):75–90. doi: 10.1534/g3.112.004283 (PMC3538345; doi:10.1534/g3.112.004283)
Supplement: Supporting Information [file supp_3.1.75_FigureS5.pdf]

A

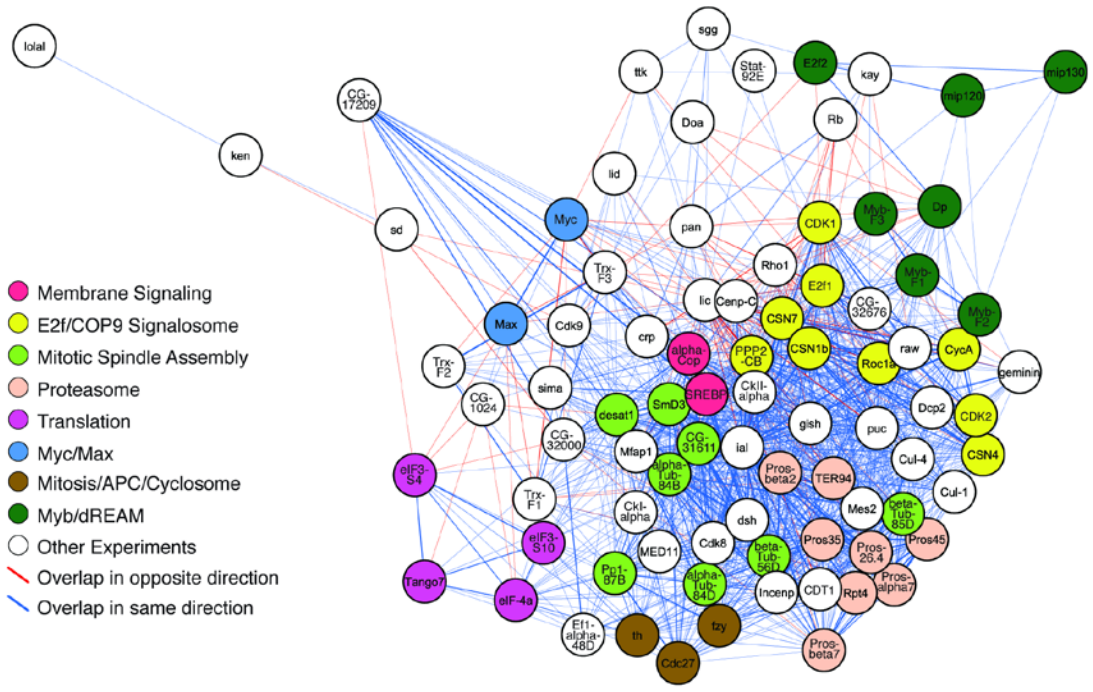

B

Rank	Gene ID	FBgn	Enrichment score	Peptides in S2 lysate	PEPTIDES IDENTIFIED Replicate 1	Replicate 2
1	lolal	FBgn0022238	8.85	13	47	68
2	RpS14a	FBgn0004403	2.00	ND	0	1
3	RpS19a	FBgn0010412	2.00	ND	0	1
4	alphaTub84B	FBgn0003884	1.33	12	7	9
5	ana3	FBgn0033718	1.00	1	0	1
6	ken	FBgn0011236	1.00	4	0	4
7	CG9791	FBgn0037232	1.00	2	2	0
8	CG2064	FBgn0033205	0.58	19	6	5
9	CG11377	FBgn0031217	0.57	7	0	4
10	ttk	FBgn0003870	0.33	6	0	2

Figure S5 (A) Network representation of the overlap between target genes that were significantly regulated in the different RNAi treatments. Overlap exceeding that expected by random selection of genes was determined for each RNAi treatment pair. Gene sets regulated in the same and opposite directions were considered separately, and the direction displaying higher overlap was selected. Nodes representing samples are connected to each other by an edge if they have excess overlap, see Methods for details). Thickness of the edge represents the level of overlap, and color indicates whether the overlapping genes were regulated in the same, blue or opposite, red direction. Network is laid out using Cytoscape, Shannon et al., 2003 yFiles organic layout algorithm. Nodes are colored according to the color scheme from **Figure 2**. Note that a different bioinformatic method used here results in a very similar classification of the samples. Overlap analysis also classifies samples that have very few regulated genes, e.g. *E2f2*; dark green and identifies connections between proteins that are found in different complexes, such as the dREAM complex, dark green that consists of *mip120*, *mip130* and either *Myb* or *E2f2*/*DP*. A heatmap of the same data classified by hierarchical clustering is shown in **Figure S4**. (B) LTQ mass-spec. Lolal protein was purified using tandem affinity purification from stable S2 cell line expressing relatively low levels of lolal protein tagged with streptavidin binding protein and hexahistidine tags, Turunen et al. 2012, in preparation). Proteins are ranked by enrichment ratio, number of peptides in sample divided by number of peptides in S2 cell lysate;, Brunner et al., 2007)). The Ken protein was among the ten most enriched proteins shown.
