# Supplementary material for: Transcriptional Networks Controlling the Cell Cycle
Source: G3 (Bethesda). 2013 Jan 1;3(1):75–90. doi: 10.1534/g3.112.004283 (PMC3538345; doi:10.1534/g3.112.004283)
Supplement: Supporting Information [file supp_3.1.75_FigureS6.pdf]

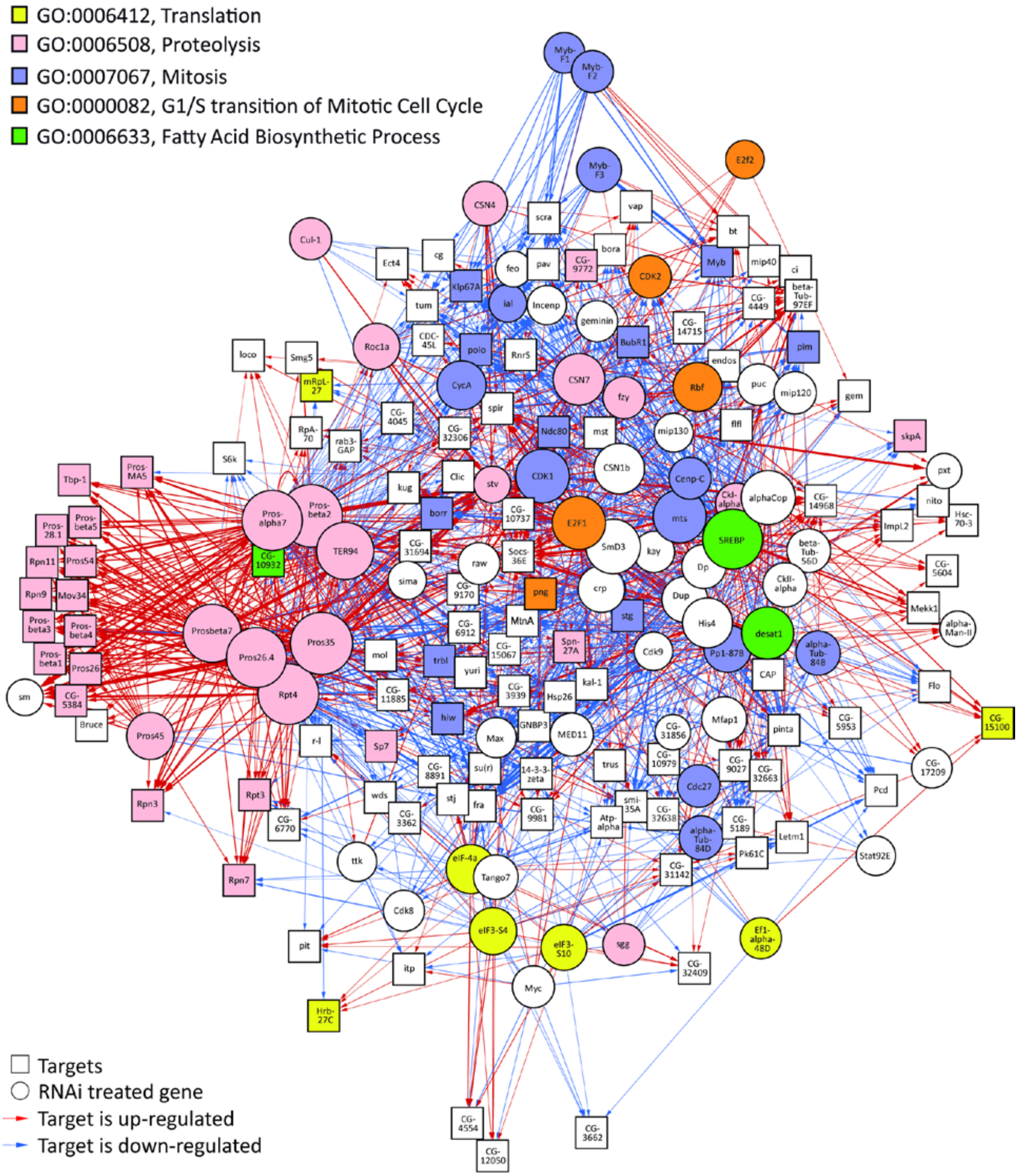

Figure S6 Transcriptional network regulating the cell cycle. Nodes are connected by an edge if a RNAi treated gene, circle results in a significant regulation of a target gene, box). Thickness of the edge represents the magnitude of the effect, and its color indicates upregulation, red or downregulation, blue of a target gene after RNAi. Size of the circles indicates the number of target genes regulated. Nodes are colored according to GO annotations indicated in the inset. Only target genes whose loss affects cell cycle or cell size in S2 cells, Bjorklund et al., 2006); Björklund et al., in preparation are included in the network. Network is laid out using yFiles organic algorithm.
