# Supplementary material for: Transcriptional Networks Controlling the Cell Cycle
Source: G3 (Bethesda). 2013 Jan 1;3(1):75–90. doi: 10.1534/g3.112.004283 (PMC3538345; doi:10.1534/g3.112.004283)
Supplement: Supporting Information [file supp_3.1.75_FigureS7.pdf]

A

	Myt1	Mapk-Ak2	Pan (dTCF)	Lic (dMEK2)
Myt1	1			
Mapk-Ak2	0	2		
Pan (dTCF)	0	0	64	
Lic (dMEK2)	0	1	0	6

B

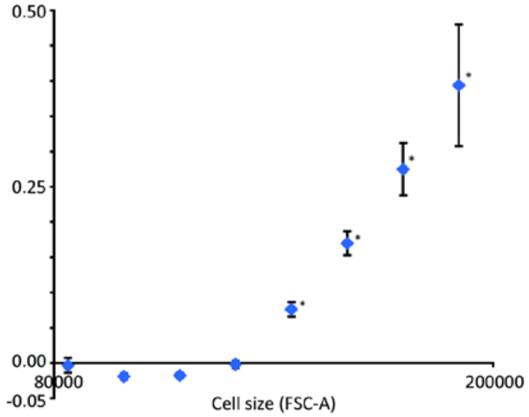

Figure S7 (A) Overlap between target genes of *Myt1*, *MAPk-Ak2*, *Pan/dTCF* and *Lic/MEK3*. Numbers indicate shared target genes. Note that no target gene is common to all of these samples. (B) Relative amount of unphosphorylated Cdk1, Cdk1-Cdk1P in *Drosophila S2* cells as a function of cell size, FSC-A). Note that unphosphorylated Cdk1 rapidly increases after the G2 cells reach a certain size. Error bars indicate one standard error, and asterisks $p < 0.01$, Kolmogorov-Smirnov test).
